# TERN-501 monotherapy and combination therapy with TERN-101 in metabolic dysfunction-associated steatohepatitis: the randomized phase 2a DUET trial

**DOI:** 10.1038/s41591-025-03722-7

**Published:** 2025-06-11

**Authors:** Mazen Noureddin, Naim Alkhouri, Eric J. Lawitz, Kris V. Kowdley, Rohit Loomba, Lois Lee, Christopher Jones, Amnon Schlegel, Tonya Marmon, Kacey Anderson, Yizhao Li, Erin Quirk, Stephen A. Harrison

**Affiliations:** 1 Houston Methodist Hospital, Houston Research Institute, Houston, TX USA; 2 Arizona Liver Health, Tucson, AZ USA; 3 Texas Liver Institute, University of Texas Health San Antonio, San Antonio, TX USA; 4 Liver Institute Northwest, Elson S. Floyd College of Medicine, Washington State University, Seattle, WA USA; 5 Division of Gastroenterology and Hepatology, MASLD Research Center, University of California San Diego, La Jolla, CA USA; 6 Terns Pharmaceuticals, Foster City, CA USA; 7 Pinnacle Clinical Research Institute, San Antonio, TX USA

**Keywords:** Drug development, Metabolic diseases

## Abstract

Thyroid hormone receptor-β (THRβ) agonism is a validated mechanism for treating metabolic dysfunction-associated steatohepatitis (MASH). DUET was a 12-week, randomized, double-blind, placebo-controlled, multicenter phase 2a study investigating the efficacy, safety and pharmacodynamics and pharmacokinetics of once-daily TERN-501 (THRβ agonist) as monotherapy or combined with TERN-101 (farnesoid X receptor agonist), in patients with presumed MASH. Overall, 162 patients were randomized to: TERN-501 monotherapy (1 mg (*n* = 23), 3 mg (*n* = 23) or 6 mg (*n* = 22)), TERN-101 10-mg monotherapy (*n* = 24), TERN-501 (3 mg (*n* = 23) or 6 mg (*n* = 23)) plus TERN-101 10-mg combination therapy or placebo (*n* = 24). The primary endpoint was relative change from baseline at week 12 in liver fat content with TERN-501 monotherapy versus placebo, using magnetic resonance imaging proton density fat fraction (MRI-PDFF). Least squares mean (s.e.) changes from baseline at week 12 in MRI-PDFF with TERN-501 were: −15.4% (5.2%) with 1 mg, −27.5% (5.7%) with 3 mg (*P* = 0.0036) and −44.8% (5.9%) with 6 mg (*P* < 0.0001), versus −4.0% (5.4%) with placebo. The incidence of adverse events was similar with TERN-501 monotherapy or placebo. In conclusion, TERN-501 treatment resulted in dose-dependent, significant reductions from baseline in MRI-PDFF compared to placebo in patients with MASH. ClinicalTrials.gov registration: NCT05415722.

## Abstract (web summary)

In the 12-week, phase 2a DUET study, treatment with TERN-501, a thyroid hormone receptor β agonist, as monotherapy or in combination with TERN-101, a farnesoid X receptor agonist, resulted in dose-dependent reductions in liver fat content as compared to placebo in patients with presumed metabolic dysfunction-associated steatohepatitis.

## Main

Metabolic dysfunction-associated steatotic liver disease (MASLD) is the hepatic manifestation of a heterogeneous, multisystem disorder associated with liver complications, increased risk of chronic kidney disease, obesity and type 2 diabetes (T2D), and cardiovascular morbidity and mortality^1,2^. Metabolic dysfunction-associated steatohepatitis (MASH) is the inflammatory form of MASLD^3^. As MASLD progresses into MASH, patients face the risk of liver fibrosis, which may progress to cirrhosis and end-stage liver disease, necessitating transplantation^1,2,4^. MASLD and MASH are updated terminology replacing nonalcoholic fatty liver disease (NAFLD) and nonalcoholic steatohepatitis (NASH), respectively^5,6^.

Globally, MASH is reported to affect up to 5% of people^4^, including 37% of those with T2D and approximately one in three people classified as overweight or obese^7,8^. As the prevalence of metabolic diseases increases, so too does MASH^3,7^. In the United States, MASH-related cirrhosis is now the leading indication for liver transplantation in women and those ≥65 years of age, and, alongside alcohol-associated liver disease, is the leading indication overall^9–11^. Indeed, mortality risk in patients with MASH is associated strongly with liver fibrosis^12^. Additionally, patients with MASH are at high risk of hepatocellular carcinoma^2,13^.

Until recently, there were no approved treatments for MASH^14,15^. Thyroid hormone receptor-β (THRβ) is highly expressed in hepatocytes and dysregulated in MASH^16,17^. THRβ agonism promotes lipophagy, mitochondrial biogenesis and mitophagy, and increased hepatic fatty acid β-oxidation, ameliorating MASH by decreasing the burden of lipotoxic lipids and reducing hepatic fat content, while promoting favorable effects on lipid profiles^17^. In studies of patients with noncirrhotic MASH, pharmacological THRβ agonism resulted in MASH resolution and fibrosis regression^18,19^. These data led to the US Food and Drug Administration approval of the THRβ agonist resmetirom as the first approved agent for treating adults with noncirrhotic MASH with moderate-to-advanced liver fibrosis^20,21^.

Due to the multifactorial pathophysiology of MASLD and MASH, combination approaches may be necessary to maximize response to pharmacological intervention^22^. Although resmetirom monotherapy has demonstrated effective MASH resolution and fibrosis improvement in a phase 3 study^19^, there remains a need for additional THRβ agonist treatment options with favorable safety and drug–drug interaction profiles, and reduced pharmacokinetic (PK) variability.

TERN-501 is an investigational, orally administered, highly potent THRβ agonist that demonstrated high selectivity for the β isoform over the α isoform of THR in vitro^23^. In a phase 1 study of healthy participants, TERN-501 was well tolerated, increased sex hormone-binding globulin (SHBG—a marker of liver THRβ engagement) in a dose-dependent manner, and significantly reduced atherogenic serum lipids versus placebo^24^. TERN-101 is an investigational farnesoid X receptor (FXR) agonist^25^ that was well tolerated, reduced alanine aminotransferase (ALT) and liver fat content (LFC), and significantly reduced corrected T1 (cT1) relaxation time in a phase 2a study of patients with presumed MASH^26,27^.

Here we report the results of the 12-week, phase 2a DUET study that evaluated the efficacy, safety, pharmacodynamics (PD), PK and combinability of TERN-501 in noncirrhotic patients with presumed MASH. To assess the combinability of TERN-501 with other oral agents, treatment groups of patients receiving TERN-501 coadministered with TERN-101 were included.

## Results

### Study design and patients

**Fig. 1:**
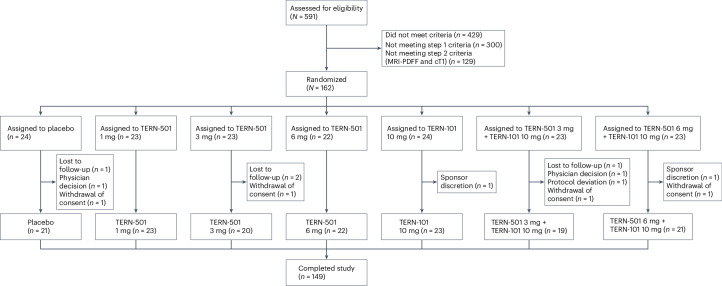
CONSORT patient flow diagram. Study overview from eligibility assessment to study completion.

From 17 June 2022 to 20 January 2023, 591 patients were screened for study eligibility and 162 underwent randomization at 27 sites to one of seven treatment groups: TERN-501 monotherapy (1 mg (*n* = 23), 3 mg (*n* = 23) or 6 mg (*n* = 22)), TERN-101 10-mg monotherapy (*n* = 24), TERN-501 (3 mg (*n* = 23) or 6 mg (*n* = 23)) in combination with TERN-101 10 mg, or placebo (*n* = 24) (Fig. 1). Forty-three patients were included in the PK/PD substudy population. Of those randomized, 149 (92%) patients completed the study; the frequencies of study withdrawal were similar between treatment groups (Fig. 1).

Patient demographics and clinical characteristics were generally well balanced across treatment groups, with some differences in rates of T2D, hypertension and statin use at baseline (Table 1). Overall, mean (s.d.) age was 53.3 (11.8) years and 54.9% were female. Most patients were white (85.8%) and were Hispanic or Latino (61.1%). Mean (s.d.) body mass index was 37.8 (6.9) kg m^−2^. The mean (s.d.) LFC measured by magnetic resonance imaging proton density fat fraction (MRI-PDFF) was 17.7% (5.5%) and mean (s.d.) cT1 relaxation time was 936.2 (98.9) ms, consistent with an at-risk MASH population^28^. Patients had a mean (s.d.) baseline ALT of 42.2 (24.1) IU l^−1^, mean (s.d.) aspartate aminotransferase (AST) of 31.2 (15.6) IU l^−1^ and mean (s.d.) fasting low-density lipoprotein cholesterol (LDL-C) of 93.9 (28.7) mg dl^−1^ (Table 1).

**Table 1:** Baseline demographics and characteristics (efficacy analysis set)

	Placebo (*N* = 24)	TERN-501 1 mg (*N* = 23)	TERN-501 3 mg (*N* = 23)	TERN-501 6 mg (*N* = 22)	TERN-101 10 mg (*N* = 24)	TERN-501 3 mg + TERN-101 10 mg (*N* = 23)	TERN-501 6 mg + TERN-101 10 mg (*N* 23)	Total (*N* = 162)
**Age, years, mean (s.d.)**	52.2 (10.5)	52.3 (13.3)	52.2 (14.4)	52.2 (11.7)	53.6 (11.8)	55.6 (12.5)	55.3 (8.4)	53.3 (11.8)
**Sex,** ***n*** **(%)**								
Male	9 (37.5)	12 (52.2)	10 (43.5)	6 (27.3)	12 (50.0)	12 (52.2)	12 (52.2)	73 (45.1)
Female	15 (62.5)	11 (47.8)	13 (56.5)	16 (72.7)	12 (50.0)	11 (47.8)	11 (47.8)	89 (54.9)
**Race,** ***n*** **(%)**								
White	22 (91.7)	20 (87.0)	22 (95.7)	18 (81.8)	19 (79.2)	20 (87.0)	18 (78.3)	139 (85.8)
Black or African American	0	1 (4.3)	0	3 (13.6)	2 (8.3)	3 (13.0)	3 (13.0)	12 (7.4)
American Indian or Alaska Native	0	2 (8.7)	1 (4.3)	0	1 (4.2)	0	1 (4.3)	5 (3.1)
Native Hawaiian or Other Pacific Islander	0	0	0	1 (4.5)	1 (4.2)	0	0	2 (1.2)
Asian	0	0	0	0	0	0	1 (4.3)	1 (0.6)
Other (Puerto Rican)	0	0	0	0	1 (4.2)	0	0	1 (0.6)
Not reported	1 (4.2)	0	0	0	0	0	0	1 (0.6)
Unknown	1 (4.2)	0	0	0	0	0	0	1 (0.6)
**Ethnicity,** ***n*** **(%)**								
Hispanic or Latino	19 (79.2)	17 (73.9)	13 (56.5)	15 (68.2)	10 (41.7)	11 (47.8)	14 (60.9)	99 (61.1)
Not Hispanic or Latino	5 (20.8)	6 (26.1)	10 (43.5)	7 (31.8)	14 (58.3)	12 (52.2)	9 (39.1)	63 (38.9)
**Body mass index** **(kg** **m**^**−2**^**), mean (s.d.)**	36.6 (6.7)	37.5 (7.8)	37.0 (7.7)	39.0 (7.5)	36.9 (4.0)	39.3 (7.9)	38.2 (6.4)	37.8 (6.9)
**LFC (%) by MRI-PDFF**								
*n*	24	23	23	22	24	23	23	162
Mean (s.d.)	17.0 (5.2)	16.6 (5.2)	19.5 (5.8)	17.3 (5.8)	17.9 (5.4)	18.8 (6.6)	16.9 (4.2)	17.7 (5.5)
**cT1 (ms)**								
*n*	24	23	23	22	24	23	23	162
Mean (s.d.)	937.3 (102.4)	921.3 (82.9)	927.6 (80.9)	920.0 (79.1)	962.2 (113.1)	977.1 (128.7)	905.8 (84.9)	936.2 (98.9)
**ALT** **(IU** **l**^**−1**^**)**								
*n*	24	23	23	22	24	23	23	162
Mean (s.d.)	43.7 (19.3)	42.0 (24.6)	39.4 (22.0)	38.2 (23.8)	39.0 (15.8)	43.0 (28.0)	50.0 (32.7)	42.2 (24.1)
**AST** **(IU** **l**^**−1**^**)**								
*n*	24	23	23	22	24	23	23	162
Mean (s.d.)	34.0 (16.8)	30.6 (14.3)	28.9 (13.2)	26.2 (9.2)	31.1 (11.8)	31.1 (16.9)	36.2 (23.2)	31.2 (15.6)
**GGT** **(IU** **l**^**−1**^**)**								
*n*	24	23	23	22	24	23	23	162
Mean (s.d.)	51.8 (26.1)	38.4 (20.8)	39.9 (24.4)	33.0 (11.2)	48.8 (32.2)	50.2 (40.5)	45.7 (26.0)	44.1 (27.6)
**Fasting LDL-C** **(mg** **dl**^**−1**^**)**								
*n*	23	22	23	22	21	20	22	153
Mean (s.d.)	87.3 (29.4)	101.6 (31.9)	101.7 (26.6)	98.8 (29.9)	84.9 (27.4)	89.4 (30.1)	93.0 (24.0)	93.9 (28.7)
**Fasting glucose** **(mg** **dl**^**−1**^**)**								
*n*	24	23	23	22	24	23	23	162
Mean (s.d.)	117.5 (36.1)	114.4 (26.8)	119.1 (37.5)	107.2 (27.2)	121.3 (42.5)	129.3 (50.6)	133.2 (43.2)	120.3 (38.7)
**T2D,** ***n*** **(%)**	11 (45.8)	8 (34.8)	10 (43.5)	6 (27.3)	7 (29.2)	13 (56.5)	13 (56.5)	68 (42.0)
**Hypertension,** ***n*** **(%)**	15 (62.5)	10 (43.5)	14 (60.9)	13 (59.1)	15 (62.5)	17 (73.9)	15 (65.2)	99 (61.1)
**Dyslipidemia,** ***n*** **(%)**	14 (58.3)	10 (43.5)	10 (43.5)	13 (59.1)	16 (66.7)	12 (52.2)	13 (56.5)	88 (54.3)
**Baseline statin use,** ***n*** **(%)**	15 (62.5)	8 (34.8)	13 (56.5)	13 (59.1)	13 (54.2)	18 (78.3)	13 (56.5)	93 (57.4)
**Baseline PCSK9 inhibitor use,** ***n*** **(%)**	2 (8.3)	0	1 (4.3)	0	0	2 (8.7)	0	5 (3.1)
**Baseline GLP-1 analog use,** ***n*** **(%)**	2 (8.3)	2 (8.7)	2 (8.7)	1 (4.5)	3 (12.5)	4 (17.4)	3 (13.0)	17 (10.5)
**Baseline levothyroxine use,** ***n*** **(%)**	1 (4.2)	0	4 (17.4)	3 (13.6)	1 (4.2)	2 (8.7)	3 (13.0)	14 (8.6)

PCSK9, proprotein convertase subtilisin/kexin type 9.

### Efficacy

**Fig. 2:**
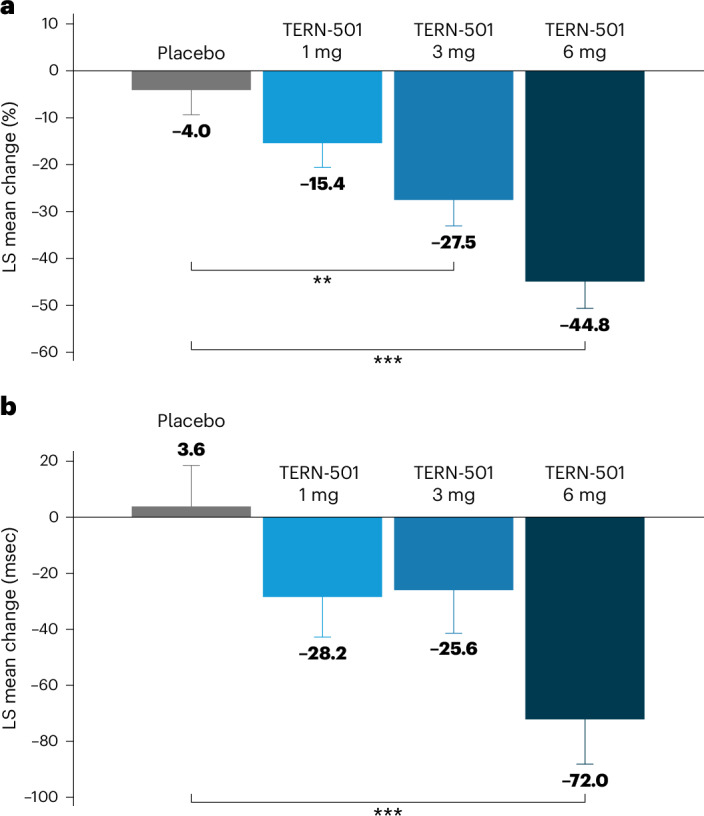
Relative changes from baseline in MRI-PDFF and cT1 relaxation time at week 12 for TERN-501 monotherapy groups (efficacy analysis set). **a**,**b**, Relative change from baseline in MRI-PDFF (**a**, placebo, *n* = 21; TERN-501 1 mg, *n* = 23 (*P* = 0.1303); TERN-501 3 mg, *n* = 19 (*P* = 0.0036); TERN-501 6 mg, *n* = 18 (*P* < 0.0001)) and cT1 relaxation time (**b**, placebo, *n* = 21; TERN-501 1 mg, *n* = 22 (*P* = 0.1289); TERN-501 3 mg, *n* = 19 (*P* = 0.1790); TERN-501 6 mg, *n* = 18 (*P* = 0.0008) at week 12 for TERN-501 monotherapy groups. Statistical analysis was performed using a type III sum of squares ANCOVA model with treatment group as a fixed effect and baseline value as the covariate; comparison between groups was conducted at the two-sided, 0.05 level of significance. No adjustments for multiplicity were made. **P < 0.01; ***P < 0.001 for TERN-501 monotherapy versus placebo. Error bars, s.e. Source data

**Fig. 3:**
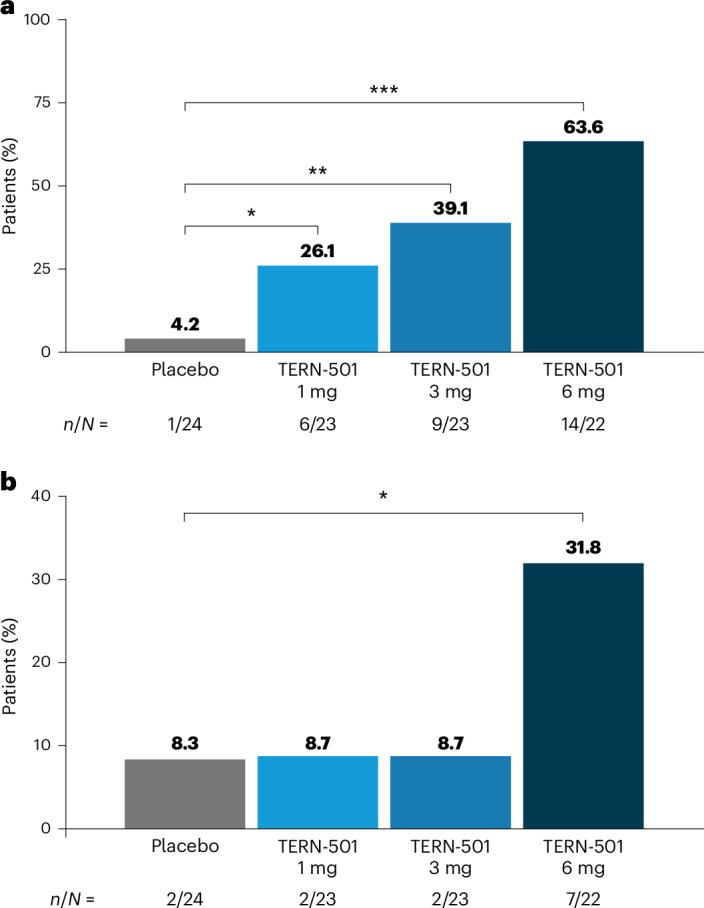
Relative reduction in liver fat and cT1 at week 12 for TERN-501 monotherapy groups (efficacy analysis set). **a**,**b**, Percentage of patients with ≥30% relative liver fat reduction (**a**, TERN-501 1 mg, *P* = 0.0349; TERN-501 3 mg, *P* = 0.0034; TERN-501 6 mg, *P* < 0.0001) and ≥80-ms reduction in cT1 (**b**, TERN-501 1 mg, *P* = 0.9645; TERN-501 3 mg, *P* = 0.09645; TERN-501 6 mg, *P* = 0.0449) at week 12 for TERN-501 monotherapy groups. *P* values were obtained using a chi-square test. Comparison between groups was conducted at the two-sided, 0.05 level of significance. No adjustments for multiplicity were made. *P < 0.05; **P < 0.01; ***P < 0.001 for TERN-501 monotherapy versus placebo. Source data

**Extended Data Fig. 1:**
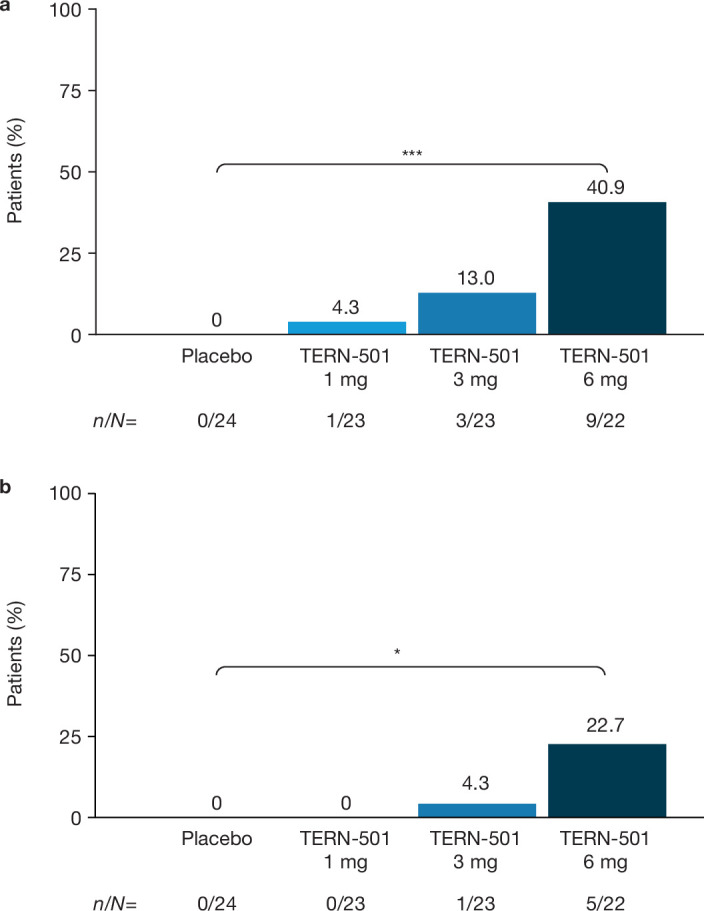
(a) Percentage of patients with ≥50% liver fat reduction and (b) percentage of patients achieving liver fat normalization at Week 12 for TERN-501 monotherapy groups (Efficacy Analysis Set). *P < 0.05; ***P < 0.001 for TERN-501 monotherapy versus placebo. Normalization of liver fat equates to magnetic resonance imaging proton density fat fraction <5%. Percentage of patients with ≥50% liver fat reduction: TERN-501 1 mg *P* = 0.3018; TERN-501 3 mg *P* = 0.0675; TERN-501 6 mg *P* = 0.0005. Percentage of patients achieving liver fat normalization: TERN-501 6 mg *P* = 0.0134. *P*-values obtained using a chi-square test. Comparison between groups was conducted at the 2-sided, 0.05 level of significance, and no adjustments for multiplicity were made. Source data

**Extended Data Fig. 2:**
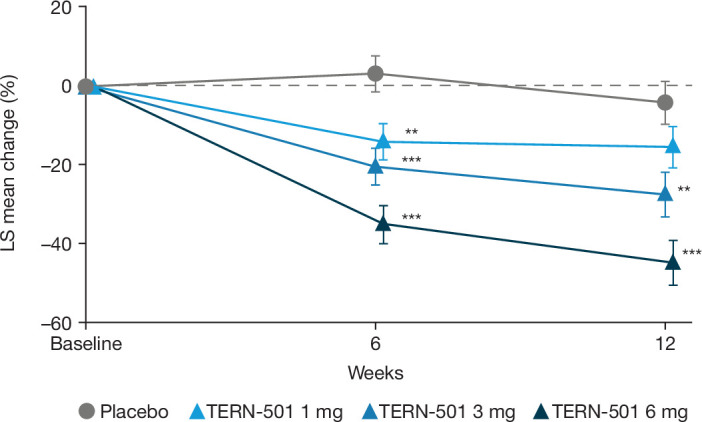
Relative change from baseline in MRI-PDFF across time for TERN-501 monotherapy groups (Efficacy Analysis Set). **P < 0.01; ***P < 0.001 for TERN-501 monotherapy versus placebo. Error bars represent standard error. Week 6: placebo (*n* = 21); TERN-501 1 mg (*n* = 23) *P* = 0.0088; TERN-501 3 mg (*n* = 22) *P* = 0.0006; TERN-501 6 mg (*n* = 20) *P* < 0.0001. Week 12: placebo (*n* = 21); TERN-501 1 mg (*n* = 23) *P* = 0.1303; TERN-501 3 mg (*n* = 19) *P* = 0.0036; TERN-501 6 mg (*n* = 18) *P* < 0.0001.Statistical analysis was performed using a type III sum of squares ANCOVA model with treatment group as a fixed effect and baseline value as the covariate; comparison between groups was conducted at the 2-sided, 0.05 level of significance, and no adjustments for multiplicity were made. LS, least squares; MRI-PDFF, magnetic resonance imaging proton density fat fraction. Source data

The primary endpoint of the study was met, with significant relative changes from baseline in MRI-PDFF at week 12 for TERN-501 monotherapy versus placebo (Fig. 2 and Supplementary Table 1). The least squares (LS) mean (s.e.) changes from baseline in MRI-PDFF were: −15.4% (5.2%), −27.5% (5.7%) and −44.8% (5.9%) with TERN-501 1 mg, 3 mg and 6 mg, respectively, versus −4.0% (5.4%) with placebo (Fig. 2 and Supplementary Table 1). The LS mean differences were statistically significant compared to placebo for the TERN-501 3-mg (*P* = 0.0036) and 6-mg (*P* < 0.0001) treatment groups (Supplementary Table 1). The percentages of patients with a response of ≥30% relative reduction in LFC at week 12 were significantly greater for all TERN-501 monotherapy groups compared to placebo (TERN-501 1 mg, 26.1% (*P* = 0.0349); 3 mg, 39.1% (*P* = 0.0034); 6 mg, 63.6% (*P* < 0.0001); placebo, 4.2%) (Fig. 3). Additionally, 40.9% of patients in the TERN-501 6-mg group achieved ≥50% reductions in LFC from baseline at week 12 versus 0% in the placebo group (*P* = 0.0005) (Extended Data Fig. 1). LFC of <5%, equating to normalization of liver fat, was achieved by 22.7% of patients in the TERN-501 6-mg group at week 12 versus 0% of patients who received placebo (*P* = 0.0134) (Extended Data Fig. 1). Notably, significant reductions in LFC were observed as early as week 6 in the TERN-501 monotherapy groups compared to placebo (1 mg, *P* = 0.0088; 3 mg, *P* = 0.0006; 6 mg, *P* < 0.0001) (Extended Data Fig. 2).

**Extended Data Fig. 3:**
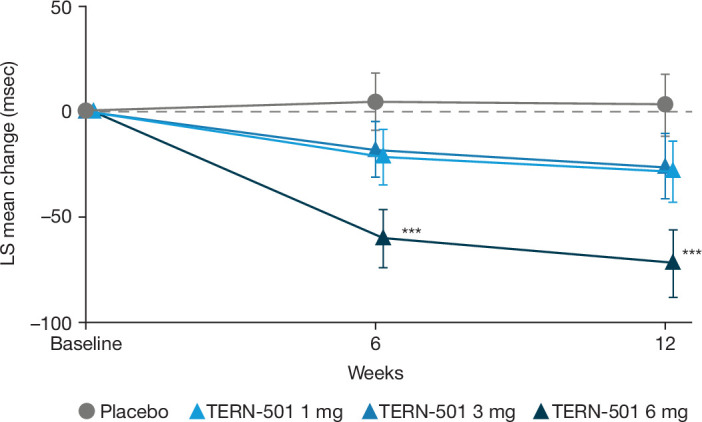
Change from baseline in cT1 relaxation time across time for TERN-501 monotherapy groups (Efficacy Analysis Set). ***P < 0.001 for TERN-501 monotherapy versus placebo. Error bars represent standard error. Week 6: placebo (*n* = 21); TERN-501 1 mg (*n* = 22) *P* = 0.1629; TERN-501 3 mg (*n* = 22) *P* = 0.2305; TERN-501 6 mg (*n* = 20) *P* = 0.0010. Week 12: placebo (*n* = 21); TERN-501 1 mg (*n* = 22) *P* = 0.1289; TERN-501 3 mg (*n* = 19) *P* = 0.1790; TERN-501 6 mg (*n* = 18) *P* = 0.0008. Statistical analysis was performed using a type III sum of squares ANCOVA model with treatment group as a fixed effect and baseline value as the covariate; comparison between groups was conducted at the 2-sided, 0.05 level of significance, and no adjustments for multiplicity were made. cT1, corrected T1; LS, least squares. Source data

All predefined secondary endpoints were met. cT1 relaxation time—a marker of fibroinflammation in the liver—decreased significantly from baseline in the TERN-501 6-mg monotherapy group at week 12 (LS mean (s.e.) change: −72.0 (16.1) ms) versus placebo (3.6 (14.9) ms; *P* = 0.0008) (Fig. 2 and Supplementary Table 2). At week 12, significantly more patients who received TERN-501 6 mg achieved a response of ≥80-ms reduction in cT1 from baseline compared to placebo (31.8% versus 8.3%, *P* = 0.0449) (Fig. 3). Additionally, by week 6, significant reductions from baseline in cT1 with TERN-501 6 mg were observed compared to placebo (*P* = 0.001) (Extended Data Fig. 3).

**Extended Data Fig. 4:**
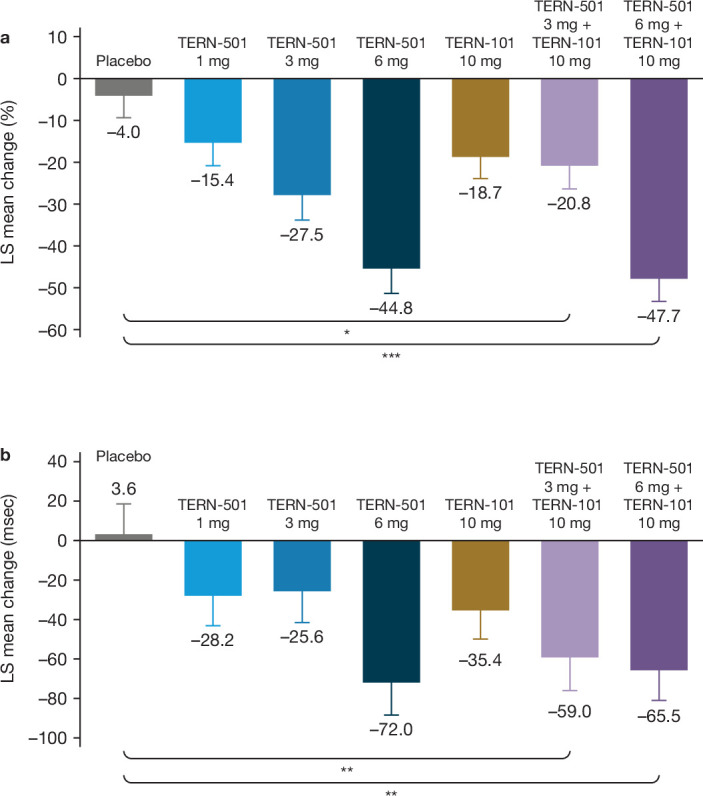
(a) Relative change from baseline (%) in MRI-PDFF and (b) change from baseline in cT1 relaxation time (msec) at Week 12 for TERN-501 plus TERN-101 combination therapy groups (Efficacy Analysis Set). *P < 0.05; **P < 0.01; ***P < 0.001 for TERN-501 plus TERN-101 combination therapy groups versus placebo. Error bars represent standard error. Relative change from baseline in MRI-PDFF: placebo (*n* = 21); TERN-101 10 mg, (*n* = 23) *P* = 0.0526; TERN-501 3 mg + TERN-101 10 mg (*n* = 19) *P* = 0.0358; TERN-501 6 mg + TERN-101 10 mg (*n* = 21) *P* < 0.0001. Change from baseline in cT1 relaxation time: placebo (*n* = 21); TERN-101 10 mg, (*n* = 23) *P* = 0.0628; TERN-501 3 mg + TERN-101 10 mg (*n* = 18) *P* = 0.0053; TERN-501 6 mg + TERN-101 10 mg (*n* = 21) *P* = 0.0014. *P* values for monotherapy groups versus placebo provided in Fig. 2. Statistical analysis was performed using a type III sum of squares ANCOVA model with treatment group as a fixed effect and baseline value as the covariate; comparison between groups was conducted at the 2-sided, 0.05 level of significance, and no adjustments for multiplicity were made. cT1, corrected T1; LS, least squares; MRI-PDFF, magnetic resonance imaging proton density fat fraction. Source data

In the TERN-501 plus TERN-101 combination therapy groups, LFC was reduced significantly from baseline at week 12 versus placebo (LS mean (s.e.) changes from baseline: −20.8% (5.7%), *P* = 0.0358 and −47.7% (5.4%), *P* < 0.0001, for TERN-501 3 mg and 6 mg plus TERN-101, respectively, versus −4.0% (5.4%) with placebo) (Extended Data Fig. 4 and Supplementary Table 1). Significant reductions in cT1 from baseline were also achieved at week 12 with TERN-501 3 mg and 6 mg in combination with TERN-101, compared to placebo (*P* = 0.0053 and *P* = 0.0014, respectively) (Extended Data Fig. 4).

### Pharmacodynamics evaluation

**Fig. 4:**
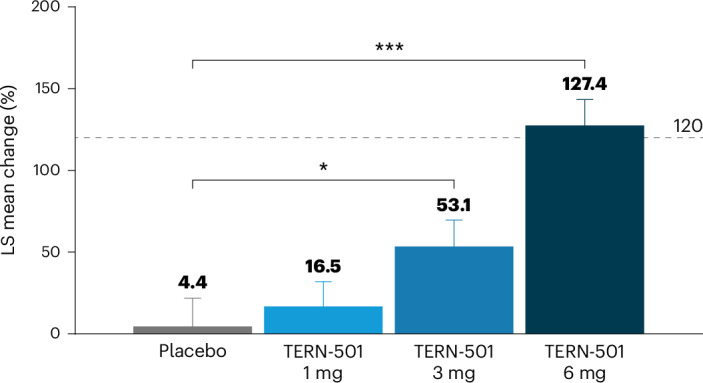
Relative change in SHBG at week 12 for TERN-501 monotherapy groups (efficacy analysis set). Relative change in SHBG at week 12 for TERN-501 monotherapy groups (placebo, *n* = 18; TERN-501 1 mg, *n* = 22 (*P* = 0.7471); TERN-501 3 mg, *n* = 19 (*P* = 0.0124); TERN-501 6 mg, *n* = 21 (*P* < 0.0001)). Dashed horizontal line at 120% represents clinically significant increases in SHBG^19^. Statistical analysis was performed using a type III sum of squares ANCOVA model with treatment group as a fixed effect and baseline value as the covariate; comparison between groups was conducted at the two-sided, 0.05 level of significance. No adjustments for multiplicity were made. Error bars, s.e. *P < 0.05; ***P < 0.001 for TERN-501 monotherapy versus placebo. Source data

SHBG increased from baseline in a dose-dependent manner at week 12 in TERN-501 monotherapy groups (LS mean (s.e.) change: 1 mg, 16.5% (15.7%); 3 mg, 53.1% (16.9%); 6 mg, 127.4% (16.1%); versus placebo, 4.4% (17.3%)) (Fig. 4) and in the TERN-501 plus TERN-101 combination therapy groups (Supplementary Table 3).

**Extended Data Fig. 5:**
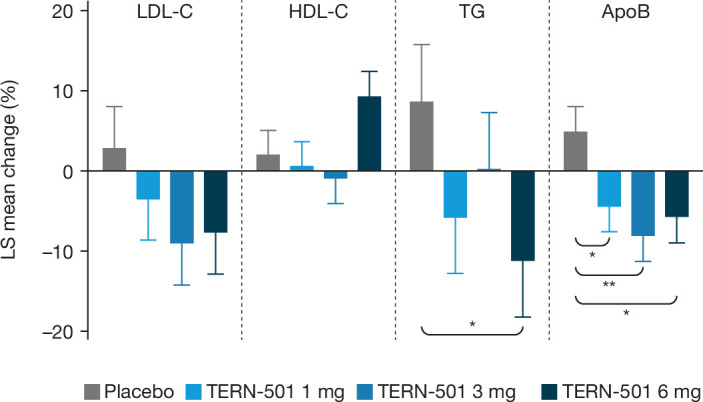
Change from baseline in lipids at Week 12 for TERN-501 monotherapy groups (Efficacy Analysis Set). *P < 0.05; **P < 0.01 for TERN-501 monotherapy versus placebo. Error bars represent standard error. *N* numbers and *P* values are provided in Supplementary Table 3. Statistical analysis was performed using a type III sum of squares ANCOVA model with treatment group as a fixed effect and baseline value as the covariate; comparison between groups was conducted at the 2-sided, 0.05 level of significance, and no adjustments for multiplicity were made. ApoB, apolipoprotein B; HDL, high-density lipoprotein; LDL, low-density lipoprotein; LS, least squares; TG, triglyceride. Source data

Treatment with TERN-501 resulted in dose-dependent changes in lipids at week 12 (Supplementary Table 3). Statistically significant relative decreases from baseline in apolipoprotein B (ApoB) were observed with TERN-501 monotherapy versus placebo (TERN-501 1 mg, *P* = 0.0389; 3 mg, *P* = 0.0051; 6 mg, *P* = 0.0206) (Extended Data Fig. 5). Additionally, decreases from baseline in total cholesterol (TC) reached significance in the TERN-501 3-mg group versus placebo (*P* = 0.0354). Decreases from baseline in triglycerides (TG) and very low-density lipoprotein cholesterol (VLDL-C) were observed in all TERN-501 monotherapy groups compared to placebo, reaching significance with TERN-501 6 mg (*P* = 0.0470 and *P* = 0.0329, respectively). Similarly, all TERN-501 monotherapy groups had relative decreases from baseline in serum lipoprotein(a) (Lp(a)) compared to increases in the placebo group; decreases were significant in the TERN-501 6-mg group (*P* = 0.0071). Relative to baseline, nonsignificant decreases in LDL-C were observed with TERN-501 monotherapy at all doses, and a nonsignificant increase in high-density lipoprotein cholesterol (HDL-C) was observed with TERN-501 6 mg. TERN-501 in combination with TERN-101 had similar effects on lipids except for HDL-C, where decreases from baseline were observed at week 12. There were significant reductions from baseline in TC and Lp(a) in both combination therapy groups versus placebo, as well as decreases in LDL-C, TG and VLDL-C (Supplementary Table 3).

### Pharmacokinetics

**Extended Data Fig. 6:**
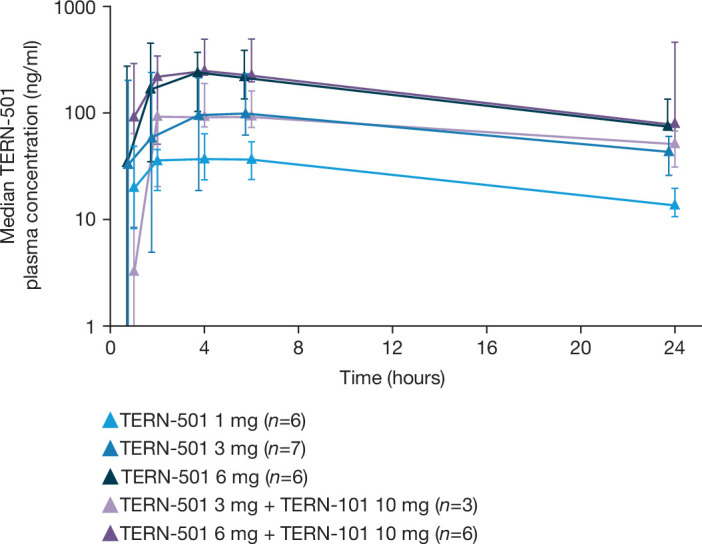
TERN-501 plasma concentration-time profile at Week 0/Day 1 (PK/PD Substudy Analysis Set). Error bars represent 95% CIs. Due to the logarithmic scale presented, 95% CIs with a zero value could not be presented and are plotted to the x-axis. CI, confidence interval. Source data

TERN-501 PK parameters were evaluated in the PK/PD substudy population. Following several doses of TERN-501, median half-life ranged from 16.0 to 19.9 h. Consistent with the elimination half-life, TERN-501 demonstrated modest (less than twofold) accumulation following several once-daily doses to steady-state across most of the dose groups. The concentration-time profile at week 0/day 1 is displayed in Extended Data Fig. 6. In general, TERN-501 demonstrated low-to-moderate PK variability across the dose ranges evaluated, and TERN-501 exposure was approximately dose proportional across the 1–6-mg dose range. TERN-501 exposures were similar when administered as monotherapy or in combination with TERN-101.

### Safety

Overall, 82 (50.6%) patients reported at least one adverse event (AE); most events were Grade 1–2. Incidences of AEs were generally similar across treatment groups and the placebo group (Table 2). The most frequent AEs, reported in ≥5% of patients in any treatment group, included pruritus (range, 0–13.6% with TERN-501 monotherapy; 4.2% with TERN-101 and 17.4–30.4% in TERN-501 plus TERN-101 combination therapy groups, versus 12.5% with placebo), headache (4.3–4.5% with TERN-501 monotherapy, 4.2% with TERN-101 and 0–8.7% in combination therapy groups, versus 8.3% with placebo) and diarrhea (4.3–21.7% with TERN-501 monotherapy, 4.2% with TERN-101 and 0–8.7% in combination therapy groups, versus 4.2% with placebo). No dose relationship was observed in these AEs (Supplementary Table 4). Three serious AEs were reported: pneumonia (Grade 3) in the TERN-501 1-mg group, cellulitis (Grade 3) in the TERN-101 10-mg group and anxiety (Grade 2) in the TERN-101 10-mg group, all of which were considered unrelated to the study drug. No deaths or Grade 4 or 5 AEs occurred. AEs leading to study drug discontinuation occurred in 0–4.5% of patients across all treatment groups. No study discontinuation occurred due to AEs (Table 2).

**Table 2:** Summary of TEAEs (safety analysis set)

AE category AE, *n* (%)	Placebo (*N* = 24)	TERN-501 1 mg (*N* = 23)	TERN-501 3 mg (*N* = 23)	TERN-501 6 mg (*N* = 22)	TERN-101 10 mg (*N* = 24)	TERN-501 3 mg + TERN-101 10 mg (*N* = 23)	TERN-501 6 mg + TERN-101 10 mg (*N* = 23)	Total (*N* = 162)
**Any TEAE**	11 (45.8)	11 (47.8)	13 (56.5)	11 (50.0)	10 (41.7)	14 (60.9)	12 (52.2)	82 (50.6)
≥Grade 3 severity^a^	0	1 (4.3)	0	0	1 (4.2)	0	0	2 (1.2)
Any serious TEAE	0	1 (4.3)	0	0	2 (8.3)	0	0	3 (1.9)
Leading to death	0	0	0	0	0	0	0	0
Leading to study drug interruption	1 (4.2)	1 (4.3)	0	1 (4.5)	1 (4.2)	1 (4.3)	0	5 (3.1)
Leading to study drug discontinuation	1 (4.2)	0	1 (4.3)	1 (4.5)	0	1 (4.3)	1 (4.3)	5 (3.1)
Leading to discontinuation from the study	0	0	0	0	0	0	0	0
**Any TEAE by maximum grade**^**a**^								
Grade 1	7 (29.2)	7 (30.4)	6 (26.1)	6 (27.3)	3 (12.5)	8 (34.8)	11 (47.8)	48 (29.6)
Grade 2	4 (16.7)	3 (13.0)	7 (30.4)	5 (22.7)	6 (25.0)	6 (26.1)	1 (4.3)	32 (19.8)
Grade 3	0	1 (4.3)	0	0	1 (4.2)	0	0	2 (1.2)
Grade 4	0	0	0	0	0	0	0	0
Grade 5	0	0	0	0	0	0	0	0
**Any study treatment-related TEAE**	5 (20.8)	1 (4.3)	4 (17.4)	4 (18.2)	2 (8.3)	6 (26.1)	4 (17.4)	26 (16.0)
≥Grade 3 severity	0	0	0	0	0	0	0	0
Any serious TEAE	0	0	0	0	0	0	0	0

A TEAE is any AE with a start date on or after the date of first administration of study drug up until 30 days after the last administration of study drug or through the follow-up period (week 16).

^a^AEs were assigned a severity grade using the Common Terminology Criteria for Adverse Events v.5.0. Medical Dictionary for Regulatory Activities v.25.1.

There were no Grade ≥3, or serious, treatment-related AEs (TRAEs) (Table 2). TRAEs reported in more than one patient in any treatment group were pruritus (7.4% overall) and diarrhea (4.3% overall), occurring at similar rates across treatment groups and placebo. Rates of gastrointestinal TRAEs were also low and similar across treatment groups. No cardiovascular TRAEs were reported.

**Extended Data Fig. 7:**
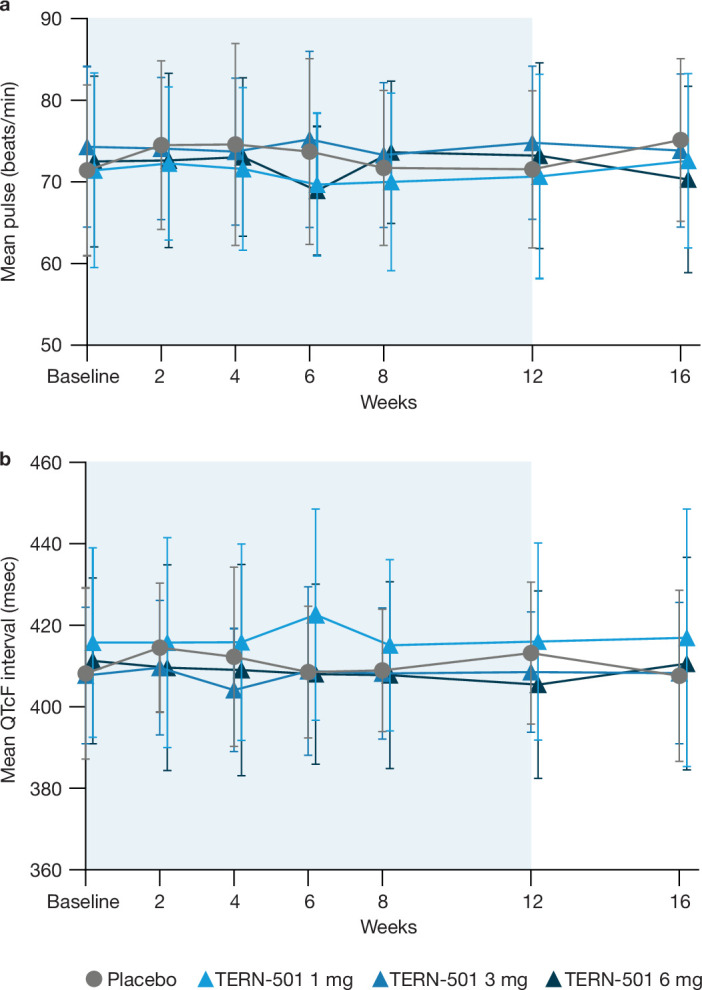
(a) Heart rate and (b) QTcF over time for TERN-501 monotherapy groups (Safety Analysis Set). Blue shaded area indicates treatment period. Error bars represent standard deviation. QTcF, QT interval corrected using Fridericia’s formula. Source data

**Extended Data Fig. 8:**
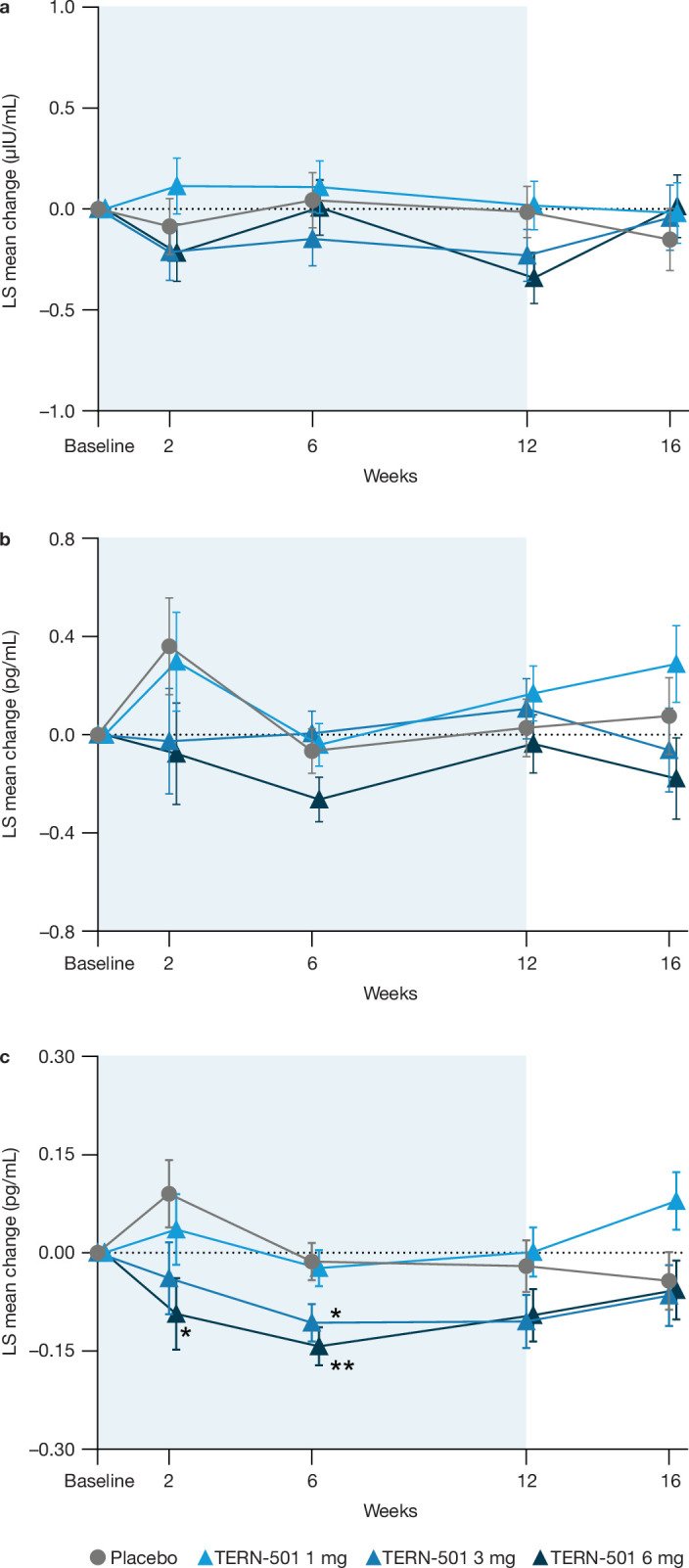
Change from baseline in (a) TSH, (b) fT3, and (c) fT4 over time for TERN-501 monotherapy groups (Safety Analysis Set). *P < 0.05; **P < 0.01 for TERN-501 monotherapy versus placebo. Blue shaded area indicates treatment period. Error bars represent standard error. Sample sizes and *P* values for each timepoint are provided in the Source Data file. Statistical analysis was performed using a type III sum of squares ANCOVA model with treatment group as a fixed effect and baseline value as the covariate; comparison between groups was conducted at the 2-sided, 0.05 level of significance, and no adjustments for multiplicity were made. LS, least squares; fT3, free triiodothyronine; fT4, free thyroxine; TSH, thyroid-stimulating hormone. Source data

**Extended Data Fig. 9:**
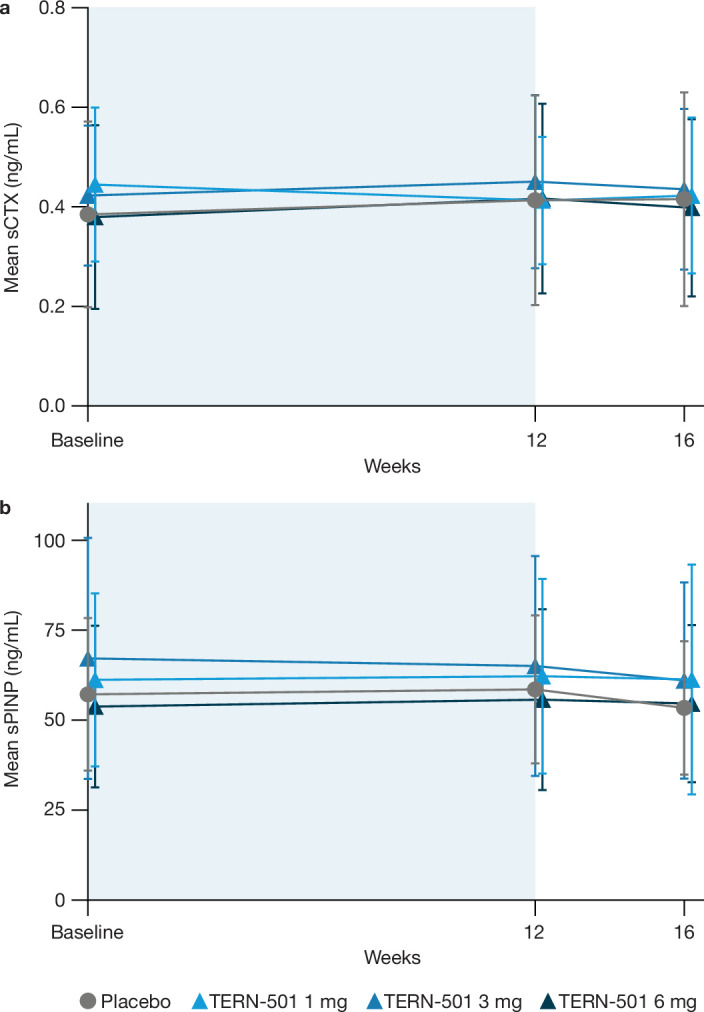
(a) sCTX and (b) sPINP over time for TERN-501 monotherapy groups (Safety Analysis Set). Blue shaded area indicates treatment period. Error bars represent standard deviation. sCTX, serum terminal telopeptide cross-link of type 1 collagen; sPINP, serum procollagen type I N-propeptide. Source data

Overall, no cardiovascular safety signals were observed in any treatment group. No clinically significant changes in vital signs, including heart rate or blood pressure, were noted in any treatment group during the study. No electrocardiogram changes were considered clinically significant by the investigator. Mean heartrates and corrected QT intervals were stable during the treatment period (Extended Data Fig. 7). No evidence of hepatobiliary or cholestatic liver injury was observed. No significant changes from baseline to week 12 were observed in thyroid-stimulating hormone (TSH) or free triiodothyronine (fT3) for all TERN-501 monotherapy and combination therapy groups compared to placebo (Extended Data Fig. 8). Dose-dependent, clinically insignificant decreases in free thyroxine (fT4) were seen at weeks 2 and 6 in the TERN-501-containing treatment groups (Extended Data Fig. 8). By week 12, there were no significant differences in fT4 for the TERN-501 monotherapy or combination therapy groups compared to placebo (Extended Data Fig. 8). No meaningful changes in free testosterone, estradiol, follicle-stimulating hormone or luteinizing hormone were observed across treatment groups in either sex (Supplementary Table 5). Glucose over time is provided in Supplementary Table 6; no changes in weight were seen across treatment groups. No significant changes from baseline in serum bone turnover markers (serum terminal telopeptide cross-link of type 1 collagen (sCTX) and serum procollagen type I N-propeptide (sPINP), markers of bone resorption and formation, respectively) were observed across the TERN-501 groups (Extended Data Fig. 9).

### Exploratory efficacy analyses

Changes from baseline at week 12 in exploratory efficacy biomarkers are shown in Supplementary Table 7. Reductions from baseline in ALT levels were observed in patients treated with TERN-501 3 mg and 6 mg, but were not statistically significant compared to placebo. Statistically significant decreases from baseline in controlled attenuation parameter (CAP) were observed in the TERN-501 3-mg and 6-mg monotherapy groups versus placebo (*P* = 0.0086 and *P* = 0.0057, respectively). Transient elastography (TE) and FibroScan-AST (FAST) score decreased nonsignificantly in all TERN-501 monotherapy and combination therapy groups. Additionally, decreases from baseline in reverse triiodothyronine (rT3) were observed in all TERN-501 monotherapy and combination therapy groups; changes were significant versus placebo in the TERN-501 6-mg group (*P* = 0.0111) and TERN-501 6-mg plus TERN-101 combination therapy group (*P* = 0.042), and led to increases in fT3/rT3 ratio. No significant changes were observed at week 12 in enhanced liver fibrosis (ELF) score (including procollagen 3 N-terminal propeptide, tissue inhibitor of metalloproteinase 1 and hyaluronic acid), released N-terminal propeptide of type III collagen (PRO-C3), cytokeratin 18 (CK-18) fragment M30 or CK-18 fragment M65, in any treatment group. Similarly, no significant changes were observed in fibrosis-4, NAFLD fibrosis score or AST to platelet ratio index.

## Discussion

In this phase 2a study, once-daily, orally administered TERN-501 as monotherapy or in combination with TERN-101 resulted in significant and dose-dependent reductions in LFC (measured by MRI-PDFF) and fibroinflammation (measured by cT1 relaxation time) in noncirrhotic patients with presumed MASH, meeting all primary and secondary endpoints. Patients treated with 12 weeks of TERN-501 monotherapy achieved up to a 44.8% mean relative reduction from baseline in LFC (versus 4.0% for placebo). These decreases in LFC are broadly in line with those reported in phase 3 studies of the approved THRβ agonist resmetirom in patients with MASH, who achieved reductions of 40.8–49.0% over 16 weeks, and 35.4–46.6% over 52 weeks^19,29^. Most patients (63.6%) receiving TERN-501 6 mg achieved LFC reductions of ≥30%, indicating improved odds of achieving histologic response (that is, at least a two-point reduction in the NAFLD activity score without worsening fibrosis) and MASH resolution^30^. LFC reductions of ≥30% have previously been associated with fibrosis improvement—a predictive factor in long-term survival for patients with MASH^19,30,31^. TERN-501 demonstrated a rapid onset of effect, with reductions in LFC reaching significance at week 6. Additionally, TERN-501 monotherapy led to dose-dependent, significant reductions in cT1 relaxation time. Approximately one-third of patients treated with TERN-501 6 mg experienced reductions from baseline in cT1 of ≥80 ms, which has previously been associated with a two-point decrease in NAFLD activity score with no worsening in fibrosis, indicative of a clinically significant improvement in MASH^32^. As expected from the MRI-PDFF results in this study, statistically significant decreases in CAP (an assessment of liver steatosis) were observed in TERN-501 treatment groups at week 12 relative to placebo.

Although not statistically significant compared to placebo, TE seemed to be improved by TERN-501- or TERN-101-containing treatment relative to placebo at week 12. As TE is a measure of liver stiffness for the assessment of liver fibrosis, >12 weeks of treatment with a greater sample size to account for variability may be necessary to demonstrate a statistically significant reversal of fibrosis measured by TE. Similarly, a longer trial may be needed to see significant changes in other biomarkers such as ELF, PRO-C3 (markers of fibrogenesis) and CK-18 (a marker of apoptosis)^18^. In our study, TERN-501 generated dose-dependent increases in SHBG, indicating robust THRβ target engagement in the liver. In phase 3 studies of resmetirom, higher SHBG responses correlated with the greatest reductions in LFC by MRI-PDFF, where increases of ≥120% were associated with histologic improvements in MASH^19,29^. Treatment with TERN-501 6 mg surpassed this important response threshold, achieving a mean increase in SHBG of ≥127.4%. Furthermore, in a post hoc analysis of the TERN-501 monotherapy groups, achievement of SHBG levels ≥120% was correlated closely with achieving a ≥30% reduction from baseline in relative LFC at weeks 6 and 12 (ref. ^33^).

Patients with MASH have a diminished capacity to convert prohormone fT4 to active hormone fT3, favoring conversion of fT4 to the inactive metabolite rT3, reflecting impaired THRβ signaling within the liver^17,34^. At week 12, mean changes from baseline in rT3 were decreased in all TERN-501 monotherapy and combination therapy groups, leading to increases in the fT3/rT3 ratio, suggesting that TERN-501 treatment may improve THRβ signaling and restore thyroid hormone levels within the liver in patients with MASH.

TERN-501 monotherapy demonstrated a favorable tolerability profile in a MASH population, with no safety signals identified and no dose-dependent safety findings. There were no treatment-related cardiovascular AEs, or any relevant changes from baseline for markers of thyroid or cardiovascular function or bone turnover, indicating lack of thyroid axis effect or THRα agonism with TERN-501. In the phase 3 MAESTRO-NASH study, dose-dependent diarrhea (30%) and nausea (20%), with an average duration of approximately 1 month, were reported by patients with biopsy-confirmed MASH treated with resmetirom^19,20^. Diarrhea and nausea were also the AEs reported most frequently in the phase 3 MAESTRO-NAFLD study of resmetirom in patients with MASLD and presumed MASH^29^, and the most common causes of treatment discontinuation in the resmetirom trials^20^. In DUET, gastrointestinal AEs in TERN-501 monotherapy treatment groups occurred mostly at low rates (diarrhea 0–21.7%, nausea 0–8.7%) that were generally similar to placebo and were not dose dependent, suggesting that gastrointestinal effects may not be related to the THRβ class overall. This is important for patients with MASH who may be receiving treatments for other comorbidities, such as glucagon-like peptide-1 (GLP-1) analogs for diabetes and weight management, that are associated with gastrointestinal AEs^35,36^. Notably, 17 patients (10.5%) in DUET were taking a GLP-1 analog at baseline. Our data suggest that the addition of TERN-501 to a patient’s existing treatment regimen may not result in overlapping gastrointestinal AE profiles, which are known to impact medication adherence^37,38^. Thus, TERN-501 has the potential to be a well-tolerated THRβ agonist treatment option for people living with MASH.

A novel aspect of the DUET study design was the inclusion of treatment groups evaluating the efficacy and safety of TERN-501 in combination with the FXR agonist TERN-101, which allowed us to assess the potential combinability of TERN-501 with other therapeutic agents. DUET is the first clinical study to evaluate THRβ agonism as part of a combination therapy approach in a MASH population. Of the few combination studies previously undertaken in patients with MASH, most failed to show efficacy or were not placebo controlled^22,39,40^. The FXR controls several pathways involved in the pathogenesis of MASH, postulated to be independent of, and complementary to, THRβ agonism, including bile acid synthesis and circulation, lipid and glucose metabolism, inflammation, fibrosis and gut barrier integrity^22,41^. Moreover, pharmacological FXR agonism has been demonstrated to reduce fibrosis in patients with noncirrhotic MASH^41,42^. The complementary modalities of THRβ and FXR agonism prompted our assessment of TERN-501 combined with TERN-101 in two of the DUET treatment groups. When TERN-501 was combined with TERN-101, improvements in MRI-PDFF and cT1 at week 12 were generally maintained or modestly improved compared to TERN-501 monotherapy. The addition of TERN-101 10 mg to TERN-501 3 mg or 6 mg resulted in broadly comparable changes from baseline in MRI-PDFF and cT1 relaxation time compared to TERN-501 monotherapy at the same dose. However, the short 12-week duration of the DUET study may have limited the ability to evaluate any additional beneficial effects of combining TERN-101 with TERN-501. One observation of note is the TERN-501 3-mg plus TERN-101 10-mg treatment group, which had a high improvement in cT1 with only a modest improvement in MRI-PDFF. These findings may reflect the greater sensitivity of cT1 to inflammation and overall tissue composition changes than MRI-PDFF^43^. No additional safety findings were reported when TERN-501 was administered in combination with TERN-101, and no dose modifications were required, indicating the potential of TERN-501 for coadministration with other agents being developed for MASH.

MASH is a multisystem disorder with a highly individualized and comorbid course in which the clinical burden is not confined to hepatic complications^1^. Patients frequently have cardiovascular disease or risk factors such as pro-atherogenic lipid profiles^1^. The clinical characteristics of patients in DUET were largely consistent with previous phase 2 studies in patients with MASH^18,44^ and reflected an at-risk, comorbid MASH population^3^. Most patients had obesity or other metabolic comorbidities, and were hypertensive, receiving statins or experiencing dyslipidemia at baseline. It is therefore noteworthy that TERN-501 treatment was associated with predominantly favorable, dose-dependent changes across lipid parameters, including significant decreases in ApoB and Lp(a), reductions of which are associated with a reduced risk of cardiovascular events^45^. Indeed, the small increases in TC and LDL-C occurring with TERN-101 therapy were mitigated by TERN-501 coadministration, supporting the lipid-lowering effects of TERN-501.

Successful pharmacological interventions for MASH must not only be well tolerated and efficacious, but also capable of integration into the clinical management of patients with a complex background of cardiometabolic comorbidity^46^. Although interactions between resmetirom and statins have been reported^20^, TERN-501 administration with concomitant background therapy was allowed in the DUET study, and over half (57%) of the DUET study population reported baseline statin use. Additionally, the PK profile of TERN-501 was predictable and showed low-to-moderate variability, with TERN-501 exposures in patients with MASH generally consistent with those previously observed in healthy participants^24^, indicating that neither MASH as a disease nor concomitant therapies being used by the patients in our trial had relevant effects on plasma concentrations of TERN-501. Therefore, TERN-501 may have the potential to avoid adverse drug interactions with commonly coprescribed medication classes in MASH.

A promising class of therapeutics for the treatment of MASH are GLP-1 receptor agonists (GLP-1RAs)^47^, which have also transformed the treatment landscape for people living with T2D and obesity based on their ability to regulate glucose and cause weight loss through reduced caloric intake, respectively^22,48^. The rapidly evolving treatment landscape in obesity may also have implications for the management of MASH, when considering the need for holistic treatment approaches^49^. Given the complementary mechanisms of action of THRβ agonists and GLP-1RAs, their combination represents a highly promising approach for the treatment of metabolic disease, of which MASH is a common comorbidity^7,50^. In a preclinical model of obesity, TERN-501 significantly enhanced the weight-loss efficacy of the GLP-1RA semaglutide and was associated with reduced metabolic adaptation—a factor that frequently limits the magnitude and sustainability of weight loss^51^. Leveraging the weight-loss benefits of GLP-1RAs and benefits of THRβ agonism on energy regulation, as well as in the liver and on lipids, may provide greater therapeutic response compared to either agent alone.

The strengths of this phase 2a study include the evaluation of the safety and efficacy of TERN-501 in participants with phenotypic MASH across a range of doses, known to produce distinct plasma concentrations as predicted by phase 1 data^24^. There are also some limitations. Our study had a small sample size that resulted in baseline imbalance in some treatment groups, including in presence of T2D, hypertension and statin use. However, data from previous THRβ agonist studies in MASH populations indicate that such differences in baseline characteristics are not expected to have impacted our study results^19,29,52,53^. Additionally, as a 12-week study, the potential to evaluate long-term changes was limited. Furthermore, due to the potential risk of elevated lipids with FXR agonism by TERN-101 (ref. ^54^), patients with LDL-C ≥ 150 mg dl^−1^ or TG > 500 mg dl^−1^ were excluded, limiting the ability to assess the effects of TERN-501 on these lipid profiles.

In summary, 12 weeks of once-daily, orally administered TERN-501 monotherapy significantly reduced LFC, and was well tolerated, with a gastrointestinal and cardiovascular safety profile similar with placebo. The overall efficacy, safety and tolerability, and PK profile of TERN-501 supports further investigation as a monotherapy or as a combination therapy for the treatment of people living with MASH.

## Methods

### Study design and patients

**Extended Data Fig. 10:**
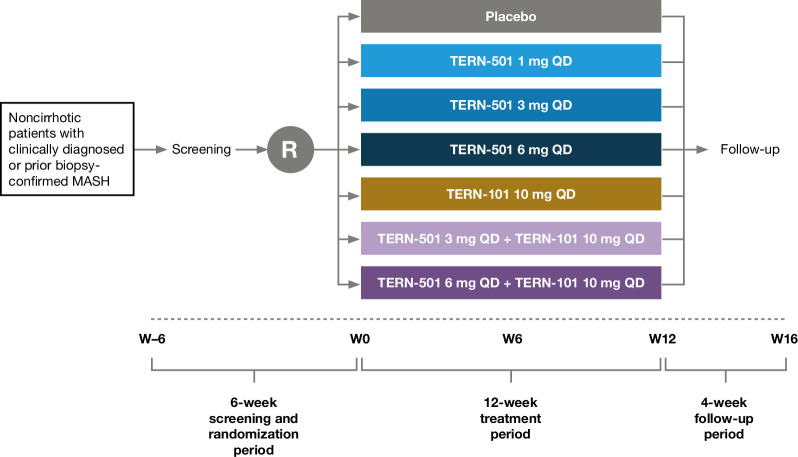
Study design. QD, once daily; R, randomization; W, Week.

DUET (NCT05415722) was a 12-week, randomized, double-blind, placebo-controlled, parallel-group, phase 2a study conducted at 35 centers (with 27 enrolling patients) across the United States (Extended Data Fig. 10). The study was conducted in accordance with the applicable International Council for Harmonisation Good Clinical Practice guidelines and the principles of the Declaration of Helsinki and Council for International Organizations of Medical Sciences International Ethical Guidelines. The protocol and subsequent amendments, and other relevant documents, were approved by the institutional review board or independent ethics committee at each study center, which are listed in the Supplementary Information.

Key inclusion criteria were: age 18–75 years, a body mass index of ≥25 kg m^−2^, glycated hemoglobin ≤9.5%, LDL-C < 150 mg dl^−1^, TG ≤ 500 mg dl^−1^ and diagnosis of noncirrhotic MASH based on previous biopsy and/or imaging criteria. A stepwise, noninvasive test-screening approach was implemented to recruit patients likely to have MASH with fibrosis, without requiring a biopsy. For step 1, where diagnosis was based on previous biopsy, patients were required to have noncirrhotic MASH with stage 1–3 fibrosis within 1 year before randomization, with no previous treatment for MASH and stable weight (<5% weight loss) since biopsy. For step 1, where diagnosis was based on imaging, patients were required to have vibration-controlled TE (VCTE) of 7.6–21 kPa and CAP > 300 dB m^−1^ by FibroScan within 3 months before screening. For step 2, all patients were required to have MRI-PDFF ≥ 10% and cT1 ≥ 800 ms.

Patients were excluded if they had evidence of chronic liver diseases other than MASLD, cirrhosis or complications of cirrhosis, severe liver impairment, previous liver transplant, current or history of thyroid disease except for patients with primary hypothyroidism who have been on stable dose of levothyroxine, or ALT or AST greater than five times the upper limit of normal. Sex was patient-reported and based on medical records. Sex and gender were not considered in the study design; sex-based analyses were not performed as the study was not powered to do so. All patients provided written informed consent before enrollment. Full eligibility criteria are described in the Supplementary Information.

### Randomization and masking

An interactive web response system was used to randomize patients in a 1:1:1:1:1:1:1 ratio to TERN-501 1 mg, TERN-501 3 mg, TERN-501 6 mg, TERN-101 10 mg, TERN-501 3 mg plus TERN-101 10 mg, TERN-501 6 mg plus TERN-101 10 mg or matching placebo. Of the 140 patients to be randomized, approximately 42 patients (about six per group) were planned to take part in an intensive PK and PD collection after the first and last dose of study drug (PK/PD substudy). Patients, investigators and study personnel were masked to treatment assignment during the study.

### Procedures

Study drugs were administered orally, once daily, for 12 weeks, with a 4-week follow-up period. Study drugs were dispensed during study visits and self-administered at home. VCTE and CAP by FibroScan were performed at screening or day 1, and at week 12. FAST score was calculated to estimate MASH risk based on VCTE and CAP combined with AST value. MRI-PDFF and cT1 were performed at screening and at weeks 6 and 12. Exploratory efficacy biomarkers, including liver enzymes (ALT, AST and gamma-glutamyl transferase (GGT)), ELF score (including 3 N-terminal propeptide, tissue inhibitor of metalloproteinase 1 and hyaluronic acid), PRO-C3, CK-18 M30 and M65, and markers of fibrosis (fibrosis-4, NAFLD fibrosis score and AST to platelet ratio index), were assessed at weeks 0, 6 and 12; TE, CAP and FAST score were assessed at weeks 0 and 12. Safety and laboratory assessments were performed at weeks 0, 2, 4, 6, 8 and 12. Follow-up safety assessments were performed at week 16. Twelve-lead electrocardiogram and clinical laboratory assessments including lipid parameters were performed at weeks 0, 2, 4, 6, 8 and 12. Thyroid hormone parameters (TSH, fT3, rT3, fT4 and fT3/rT3 ratio) were assessed at weeks 0, 2, 6 and 12. Bone turnover markers (sCTX and sPINP) were assessed at week 0 and week 12. AEs were coded using Medical Dictionary for Regulatory Activities v.25.1 and graded using the Common Terminology Criteria for Adverse Events v.5.0. Treatment-emergent adverse event (TEAEs) were defined as any AE with start date on or after the first administration of study drug through 30 days after last administration of study drug or up to week 16. All AEs reported were treatment emergent. For the PD analysis, pre-dose lipid panel assessments and SHBG were collected from all patients at weeks 0, 2, 4, 6 and 12. For the PK analysis (TERN-501), pre-dose blood samples were collected from all patients at week 0. For the PK/PD substudy, additional samples were taken at 1, 2, 4, 6 and 24 h postdose at week 0.

### Outcomes

The primary endpoint was the relative change from baseline in LFC, assessed by MRI-PDFF at week 12, for TERN-501 versus placebo. Secondary endpoints included: change from baseline in liver fibroinflammation (assessed by cT1 relaxation time) at week 12 for treatment groups receiving TERN-501, or TERN-501 in combination with TERN-101, versus placebo; and relative change from baseline in LFC assessed by MRI-PDFF at week 12 for TERN-501 plus TERN-101 combination therapy groups versus placebo. Safety endpoints included the incidence of TEAEs. Exploratory endpoints included relative change from baseline in MRI-PDFF over time, change from baseline in cT1 over time, response analyses for MRI-PDFF (≥30% relative liver fat reduction) and cT1 (≥80-ms reduction), change from baseline at week 12 in exploratory efficacy biomarkers, percentage change from baseline in SHBG and lipids at week 12 (PD analysis) and TERN-501 plasma PK parameters at week 0/day 1 and week 12.

### Statistical analysis

Data analysis was performed using SAS v.9.4. The efficacy analysis set included all randomized patients who received at least one dose of study drug, with treatment assignment based on the randomized treatment. The safety analysis set included all randomized patients who received at least one dose of study drug, with treatment assignment based on the treatment received. The PK/PD substudy population included all patients enrolled into the substudy who received at least one dose of study drug, assigned by treatment received. The optimal sample size was determined as approximately 140 patients, assigned randomly to one of the seven study groups. Based on an assumed pooled SD of 22% with a two-sided alpha of 0.05, a mean relative reduction difference in MRI-PDFF at week 12 of 23% between TERN-501 monotherapy and placebo would provide approximately 90% power, and a mean relative reduction difference in MRI-PDFF at week 12 of 36% between TERN-501 plus TERN-101 combination therapy and placebo would provide over 90% power. Based on an assumed pooled SD of 82 ms with a two-sided alpha of 0.05, a mean reduction difference in cT1 at week 12 of 77 ms between TERN-510 monotherapy and placebo would provide approximately 82% power, and a mean reduction difference in cT1 at week 12 of 134 ms between TERN-501 plus TERN-101 combination therapy and placebo would provide over 90% power.

Categorical data are presented as frequency counts and percentages. Efficacy endpoints (cT1 and MRI-PDFF analyses) used an analysis of covariance (ANCOVA) model with relative change from baseline at week 12 as the dependent variable including treatment group as a fixed effect and baseline as the covariate, and are presented as estimates of LS means, s.e. and 95% CI by treatment group. Estimates of the LS mean difference between treatment groups and placebo are presented with the associated s.e. of the difference, 95% CI of the difference and pairwise comparisons. For primary analyses, missing data were not imputed.

Analysis of other efficacy endpoints and safety parameters used a similar ANCOVA model. Exploratory efficacy biomarkers and markers of fibrosis are presented as means and s.d., with LS estimates of the mean difference, s.e., 95% CI and pairwise comparisons between each TERN active treatment group versus placebo comparison. For MRI-PDFF responders (defined as the percentage of patients with ≥30% or ≥50% relative reduction from baseline in MRI-PDFF at week 12) and cT1 responders (defined as the percentage of patients with ≥80-ms reduction from baseline in cT1 relaxation time at week 12), the number and percentage of patients in each category (response, nonresponse) are presented with a pairwise comparison of difference in percentage of responders and *P* value using a chi-squared test. To address multiple comparisons, a hierarchical testing approach was employed. Noncompartmental PK parameters were estimated from individual plasma concentration data using Phoenix WinNonlin v.8.3.5.

### Reporting summary

Further information on research design is available in the Nature Portfolio Reporting Summary linked to this article.

## Online content

Any methods, additional references, Nature Portfolio reporting summaries, source data, extended data, supplementary information, acknowledgements, peer review information; details of author contributions and competing interests; and statements of data and code availability are available at 10.1038/s41591-025-03722-7.

## Supplementary information

Supplementary Information Supplementary Notes, Investigators, Study eligibility criteria and Tables 1–7.

Reporting Summary

## Source data

Figs. 2–4 and Extended Data Figs. 1–9 Statistical source data.

## Data Availability

Datasets generated as part of the DUET study are considered sensitive and, as such, are not publicly available. Requests for data supporting the findings in this manuscript should be made to the corresponding author (M.N.: NoureddinMD@houstonresearchinstitute.com) and will be considered on a case-by-case basis 3 years after publication. Data may be shared in the form of aggregate data summaries and via a data transfer agreement with qualified noncommercial, scientific and medical researchers at the researcher’s request. Timescales vary depending on the request and may take several months after full submission of the request for sharing of the requested data or documents. Individual patient-level data are subject to patient privacy and cannot be shared. Source data are provided with this paper.
