# Supplementary material for: TERN-501 monotherapy and combination therapy with TERN-101 in metabolic dysfunction-associated steatohepatitis: the randomized phase 2a DUET trial
Source: Nat Med. 2025 Jun 11;31(7):2297–305. doi: 10.1038/s41591-025-03722-7 (PMC12283411; doi:10.1038/s41591-025-03722-7)
Supplement: Supplementary file 1 — Supplementary Notes, Investigators, Study eligibility criteria and Tables 1–7. [file 41591_2025_3722_MOESM1_ESM.pdf]

nature medicine

Supplementary information https://doi.org/10.1038/s41591-025-03722-7

TERN-501 monotherapy and combination therapy with TERN-101 in metabolic dysfunction-associated steatohepatitis: the randomized phase 2a DUET trial

In the format provided by the authors and unedited

TERN-501 monotherapy and combination therapy with TERN-101 in metabolic dysfunction-associated steatohepatitis:

the randomized phase 2a DUET trial

Mazen Noureddin[1], Naim Alkhouri[2], Eric J. Lawitz[3], Kris V. Kowdley[4], Rohit Loomba[5], Lois Lee[6], Christopher Jones[6], Amnon Schlegel[6], Tonya Marmon[6], Kacey Anderson[6], Yizhao Li[6], Erin Quirk[6], Stephen A. Harrison*[7]

[1]Houston Methodist Hospital, Houston Research Institute, Houston, TX, USA

[2]Arizona Liver Health, Tucson, AZ, USA

[3]Texas Liver Institute, University of Texas Health San Antonio, San Antonio, TX, USA

[4]Liver Institute Northwest, Elson S. Floyd College of Medicine, Washington State University, Seattle, WA, USA

[5]MASLD Research Center, Division of Gastroenterology and Hepatology, University of California San Diego, La Jolla, CA, USA

[6]Terns Pharmaceuticals, Foster City, CA, USA

[7]Pinnacle Clinical Research Institute, San Antonio, TX, USA

*Deceased

Supplementary information

Contents

TERN-501 monotherapy and combination therapy with TERN-101 in metabolic dysfunction-associated steatohepatitis: a randomized phase 2a trial....1

Supplementary information....2

Contents....2

Supplementary notes....3

Investigators....3

Study eligibility criteria....4

Inclusion criteria....4

Exclusion criteria....5

Medical conditions....5

Prior therapy....10

Prior clinical study experience....11

Supplementary tables....12

Supplementary Table 1 | Relative change from baseline in MRI-PDFF at Week 12 for all treatment groups (Efficacy Analysis Set)....12

Supplementary Table 2 | Change from baseline in cT1 at Week 12 for all treatment groups (Efficacy Analysis Set)....13

Supplementary Table 3 | Percentage change from baseline in PD markers at Week 12 for all treatment groups (Efficacy Analysis Set)....14

Supplementary Table 4 | TEAEs reported in ≥5% of patients in any treatment group by Preferred Term (Safety Analysis Set)....18

Supplementary Table 5 | Percentage change from baseline in sex hormones at Week 12 for all treatment groups (Safety Analysis Set)....19

Supplementary Table 6 | Glucose over time for all treatment groups (Safety Analysis Set)....23

Supplementary Table 7 | Change from baseline in exploratory biomarkers at Week 12 (Efficacy Analysis Set)....24

Supplementary notes

Investigators

The following investigators participated in the DUET clinical trial:

Robert A. Jenders, MD, National Research Institute, Panorama City, CA, USA

Douglas Denham, DO, Clinical Trials of Texas, LLC, San Antonio, TX, USA

Tarek Hassanein, MD, Southern California GI and Liver Centers, Coronado, CA, USA

Reem Ghalib, MD, Texas Clinical Research Institute, LLC, Arlington, TX, USA

Eric J. Lawitz, MD, Texas Liver Institute, University of Texas Health San Antonio, San Antonio, TX, USA

William E. Sanchez, MD, Floridian Clinical Research, LLC, Miami Lakes, FL, USA

Anita Kohli, MD, The Institute for Liver Health DBA Arizona Liver Health, Chandler, AZ, USA

Naim Alkhouri, MD, Arizona Liver Health, Tucson, AZ, USA

Kris V. Kowdley, MD, Liver Institute Northwest, Elson S. Floyd College of Medicine, Washington State University, Seattle, WA, USA

Raj Vuppalanchi, MBBS, Indiana University School of Medicine, Indianapolis, IN, USA

Mazen Noureddin, MD, Houston Methodist Hospital, Houston Research Institute, Houston, TX, USA

Zeid Kayali, MD, Inland Empire Clinical Trials, LLC, Rialto, CA, USA

Souvik Sarkar, MD, PhD, Florida Research Institute, Lakewood Ranch, FL, USA

Kathryn J. Lucas, MD, Lucas Research, Inc. Morehead City, NC, USA

Joseph Galati, MD, Liver Specialists of Texas, Houston, TX, USA

Paul Martin, MD, University of Miami - Miller School of Medicine, Miami, FL, USA

Jacques Benun, MD, Pinnacle Clinical Research Institute, Austin TX, USA

Rashmee Patil, MD, South Texas Research Institute, Edinburg, TX, USA

Anuj Bhargava, MD, Iowa Diabetes and Endocrinology Research Center, West Des Moines, IA, USA

Humberto Aguilar, MD, Louisiana Research Center, LLC, Shreveport, LA, USA

Alexander White, MD, Progressive Medical Research, Port Orange, FL, USA

Madhavi Rudraraju, MD, Pinnacle Clinical Research Institute, San Antonio, TX, USA

Adolfo Cueli, MD, Integrity Clinical Research, LLC, Doral, FL, USA

Yaneicy Gonzalez-Rojas, MD, Optimus U Inc., Miami, FL, USA

Joseph F. Kosinski, MD, Premier Medical Group, Clarksville, TN, USA

Rizwana Mohseni, DO, Catalina Research Institute, LLC, Montclair, CA, USA

Reed Hogan, MD, Gastrointestinal Associates and Endoscopy Center, PA – Flowood, Flowood, MS, USA

Study eligibility criteria

Inclusion criteria

Patients were eligible to be included in the study only if all the following criteria were met:

1. Must be 18–75 years of age inclusive at the time of signing the informed consent
2. Male or female, willing to follow contraception requirements defined by study protocol; female patients of childbearing potential must also have negative serum pregnancy test at screening, not be breastfeeding, and not plan to become pregnant during the study or within 30 days after dosing of study drug
3. Patients who were overweight or obese, with a body mass index ≥25 kg/m^2
4. Metabolic dysfunction-associated steatohepatitis (MASH) diagnosed by prior biopsy and/or imaging criteria as follows:
    a. For diagnosis based on prior biopsy:
        i. Step 1: noncirrhotic MASH with fibrosis (F1, F2, or F3 based on NASH Clinical Research Network scoring system) diagnosed by historical biopsy within 1 year prior to randomization, with no subsequent treatment for MASH and stable weight (<5% weight loss) since the time of the biopsy. Results from a previous study biopsy are permissible if drug was deemed nontherapeutic or patient received placebo
        ii. Step 2: patients meeting these and all other eligibility criteria will undergo magnetic resonance imaging (MRI) and must have liver fat content by proton density fat fraction (PDFF) ≥10% and corrected T1 (cT1) ≥800 msec for randomization
    b. For diagnosis based on imaging:
        i. Step 1: liver stiffness measured by transient elastography (TE) of 7.6–21 kPa and controlled attenuation parameter (CAP) >300 dB/m by FibroScan® within 3 months prior to screening (if results are available within this time frame, assessment does not need to be performed at

screening, but will be collected on Day 1. If TE and CAP were performed at screening, these do not need to be repeated on Day 1)

ii. Step 2: patients meeting these and all other eligibility criteria will undergo MRI and must have liver fat content by PDFF ≥10% and cT1 ≥800 msec for randomization

5. All patients must have MRI-PDFF ≥10% and cT1 ≥800 msec
6. Capable of giving signed informed consent, which includes compliance with the requirements and restrictions listed in the informed consent form and in this protocol, including ability to consistently take study drug once daily on an empty stomach

Exclusion criteria

Patients were excluded from the study if any of the following criteria apply:

Medical conditions

1. History or clinical evidence of chronic liver diseases other than metabolic dysfunction-associated steatotic liver disease (MASLD), including but not limited to:
    a. Active hepatitis B defined as positive Hep B surface antigen at screening
    b. Active hepatitis C defined as positive Hep C virus (HCV) antibody (anti-HCV) and HCV RNA. Patients with anti-HCV but negative HCV RNA will be eligible for participation if HCV RNA has been negative for at least 1 year
    c. Autoimmune liver disease
    d. Primary biliary cirrhosis
    e. Primary sclerosing cholangitis
    f. Wilson’s disease
    g. Gilbert’s syndrome if direct bilirubin is above 0.3 mg/dL or evidence of active hemolysis
    h. Hemochromatosis or other iron overload

i. Alpha-1-antitrypsin deficiency

j. Alcoholic liver disease

k. Prior known drug-induced hepatotoxicity

l. Known bile duct obstruction

m. Suspected or proven liver cancer

2. History or known clinical evidence of cirrhosis, esophageal varices, hepatic decompensation, or other severe liver impairment, including ascites, hepatic encephalopathy, and variceal bleeding

3. History of liver transplant or current placement on a liver transplant list

4. Current or prior thyroid cancer, thyrotoxicosis, or pituitary disorders, or with known multiple endocrine neoplasia type 2 syndrome

5. Abnormal thyroid examination including but not limited to enlarged thyroid gland (i.e., goiter) or thyroid nodules

6. Current diagnosis or history of thyroid disease, except for patients with primary hypothyroidism who have been on stable dose of thyroid hormone replacement therapy with levothyroxine for at least 6 months prior to screening through randomization; patients must have normal thyroid-stimulating hormone (TSH) and free thryroxine (fT4), per exclusion criteria #7 and be clinically euthyroid at screening

7. Clinical evidence of thyroid disease indicated by TSH or fT4 levels outside the normal range at screening

- Note: for patients in whom there is no known history of thyroid disease and who are not on thyroid hormone replacement medication, if TSH is up to 1.5 × upper limit of normal (ULN) at screening with normal fT4, one repeat test is allowed to confirm the elevation in TSH. If TSH and fT4 are normal upon repeat testing and there is no clinical suspicion of thyroid disease or history of elevated TSH, the patient may be

included. All thyroid axis testing should be performed in the morning at approximately the same time

8. Total bilirubin >1.2 mg/dL
9. Albumin <3.5 g/dL
10. International normalized ratio >1.2 in patients who are not on anticoagulant therapy
11. Alanine aminotransferase (ALT) or aspartate aminotransferase (AST) >5 × ULN
12. Unstable elevated ALT or AST: patients with ALT or AST >60 IU/L must have evidence of a stable value over at least a 2-week period prior to randomization as evidenced by one of the following:
    a. Comparison with a historical value, if available, obtained within 2–12 weeks prior to the screening visit: screening value must be ≤30% higher than a documented historical value (note: the historical value must be ≤5 × ULN)

       OR

    b. Repeat value at least 2 weeks after the screening value: repeat value should be ≤30% higher than the value from the screening visit and the repeat value must be ≤5 × ULN (note: repeat only the analyte(s) that is/are >60 IU/L at screening)

- Note: if the Investigator has clinical suspicion for acute liver injury in a patient for other reasons, the patient should be excluded after discussion with the Medical Monitor
- Note: the MRI-PDFF and cT1 may proceed based on the initial ALT value at screening, given all other eligibility criteria are met, but randomization should not occur prior to confirmation of ALT and/or AST stability as specified above

13. Alkaline phosphatase >1.5 × ULN
14. Platelet count <150,000/mm^3
15. Estimated glomerular filtration rate <60 mL/min/1.73m^2

16. Weight loss of >5% total body weight within 3 months prior to screening
17. History of a malignancy within 2 years of screening, with the following exceptions:
    - Adequately treated carcinoma in situ of the cervix
    - Adequately treated basal or squamous cell cancer or other localized nonmelanoma skin cancer
18. Prior or planned (during the study period) bariatric surgery (e.g., gastroplasty, roux-en-Y gastric bypass)
19. Type 1 diabetes
20. Uncontrolled diabetes with hemoglobin A1c >9.5%. Note: patients with or without type 2 diabetes may be enrolled provided all eligibility criteria are met
21. Unstable cardiovascular disease defined as myocardial infarction, unstable angina, percutaneous intervention, coronary artery bypass graft, or stroke within 6 months prior to randomization
22. Uncontrolled hyperlipidemia defined as fasting low-density lipoprotein cholesterol (LDL-C) ≥150 mg/dL despite treatment or triglycerides >500 mg/dL
23. LDL-C <40 mg/dL in patients who are not taking LDL-C–lowering therapy
24. Clinically significant abnormalities in 12-lead electrocardiogram (ECG) at screening, or known history of such abnormalities, including but not limited to:
    - II/III-degree atrioventricular block or bundle branch block (right and left)
    - Arrhythmia (except occasional supraventricular premature beat)
    - QT-interval corrected for heart rate using Fridericia's method (QTcF) >450 msec for males or >470 msec for females
    - Personal or family history of prolonged QT syndrome

- Note: an ECG can be repeated in instances of lead placement error, incomplete or uninterpretable tracings, or suspicion of machine error at screening

25. Known hypersensitivities to the study drugs (TERN-501 or TERN-101) or to any formulation excipients
26. Clinically significant multiple or severe drug allergies, intolerance to topical corticosteroids, or severe post-treatment hypersensitivity reactions
27. Known allergy, intolerance, or contraindication to beta blockers
28. Alcohol consumption of >2 standard drinks per day for males and >1 standard drink per day for females over a period of more than 3 consecutive months in the year prior to screening. Remote history of alcoholism with abstinence >12 months prior to screening and no history of alcoholic liver disease is permissible
    - Note: a standard drink is defined as 12 oz (360 mL) beer (5% alcohol), 1.5 oz (45 mL) 80 proof liquor (40% alcohol), or 5 oz (150 mL) wine (12% alcohol)
29. Illicit substance/chemical abuse in the 12 months prior to screening
30. Cannabis use within 14 days of randomization
31. Unwilling to abstain from excessive alcohol use (defined in exclusion criterion #28 above), cannabis use, and illicit substance use during study participation
32. Contraindications or inability to complete MRI scanning (i.e., weight restrictions, presence of permanent pacemaker, implanted cardiac devices, etc.)
33. Positive for human immunodeficiency virus infection
34. Laboratory or clinical evidence of current infection with severe acute respiratory syndrome coronavirus 2, that is, coronavirus disease 2019 (COVID-19)
35. Presence of any condition that could, in the opinion of the Investigator, compromise the patient’s ability to participate in the study

Prior therapy

36. Historical or current use of drugs or therapies that may interfere with thyroid function (except for thyroid hormone replacement therapy with levothyroxine per exclusion criteria #6 and #37), including but not limited to methimazole, propylthiouracil, tyrosine kinase inhibitors, lithium, iodide, and propranolol; these therapies are also prohibited through end of study follow-up
37. Initiation, discontinuation, or dose adjustment of thyroid hormone replacement therapy within 6 months prior to screening through randomization
    - Note: patients must be clinically euthyroid per exclusion criteria #6, with normal TSH and T4 per exclusion criteria #7)
38. Use of medications specifically for weight loss within 12 months prior to randomization; these therapies are also prohibited through end of study follow-up
39. Use within 3 months prior to screening through randomization: resmetirom or other investigational thyroid hormone receptor-β agonists, OCA or other investigational farnesoid X receptor agonists, pioglitazone or other peroxisome proliferator-activated receptor-γ agonists, or high-dose vitamin E (>400 IU/day); these therapies are also prohibited through end of study follow-up
40. Initiation, discontinuation, or dose adjustment within 3 months prior to screening (i.e., must be on stable dose for 3 months prior to screening) through randomization; or dose adjustment expected during study participation:
    - Glucagon-like peptide-1 analogs, dipeptidyl peptidase-4 inhibitors, and sodium-glucose co-transporter 2 inhibitors
    - Insulin with >30% dose adjustment
        - Note: ≤30% dose adjustment is allowed
    - Statins, PCSK9 inhibitors, or other lipid-modifying medications

- Vitamin E ≤400 IU/day
- Hormonal contraceptives

41. Use within 3 months prior to randomization:
    - Medications potentially impacting steatohepatitis, including but not limited to systemic corticosteroids (prednisone equivalent of >10 mg/day for >2 weeks), amiodarone, tamoxifen, and methotrexate; these therapies are also prohibited from randomization through end of study follow-up
        - Note: use of systemic corticosteroids for ≤2 weeks within 3 months prior to randomization and through the end of the study is allowed
42. Initiation, discontinuation, or dose titration within 2 months prior to randomization (i.e. must be on stable dose for 2 months prior to randomization); or dose adjustment expected during study participation:
    a. Metformin and other antidiabetic drugs not described above
43. Use within 2 weeks prior to randomization:
    - Vaccination for influenza, COVID-19, or other routine vaccinations
    - Strong cytochrome P450 3A (CYP3A), P-glycoprotein (P-gp), or breast cancer resistance protein (BCRP) inducers or inhibitors, or anticipated to require treatment with strong CYP3A, P-gp, or BCRP inducers or inhibitors during study participation

Prior clinical study experience

44. Participation in another clinical study within 3 months or 5 half-lives of the other investigational agent (whichever is longer) prior to randomization. Patients who can demonstrate they did not receive active drug or who received an investigational treatment that was deemed nontherapeutic during clinical study participation are eligible for enrollment

Supplementary tables

Supplementary Table 1 | Relative change from baseline in MRI-PDFF at Week 12 for all treatment groups (Efficacy Analysis Set)

	**Placebo (*N*=24)**	**TERN-501 1 mg (*N*=23)**	**TERN-501 3 mg (*N*=23)**	**TERN-501 6 mg (*N*=22)**	**TERN-101 10 mg (*N*=24)**	**TERN-501 3 mg + TERN-101 10 mg (*N*=23)**	**TERN-501 6 mg + TERN-101 10 mg (*N*=23)**
**Baseline**							
n	24	23	23	22	24	23	23
Mean (SD)	17.0 (5.2)	16.6 (5.2)	19.5 (5.8)	17.3 (5.7)	17.9 (5.4)	18.8 (6.6)	16.9 (4.2)
**Week 12**							
n	21	23	19	18	23	19	21
Mean (SD)	16.3 (5.7)	14.2 (5.8)	14.0 (6.6)	8.8 (4.1)	14.5 (5.5)	14.6 (6.8)	8.8 (3.8)
**Relative change (%) from baseline at Week 12**							
n	21	23	19	18	23	19	21
LS mean (SE)[a]	−4.0 (5.4)	−15.4 (5.2)	−27.5 (5.7)	−44.8 (5.9)	−18.7 (5.2)	−20.8 (5.7)	−47.7 (5.4)
LS mean difference (SE)[a,b]		−11.4 (7.5)	−23.5 (7.9)	−40.8 (8.0)	−14.7 (7.5)	−16.7 (7.9)	−43.7 (7.6)
95% CI for LS mean difference[a,b]		−26.2, 3.4	−39.1, −7.8	−56.5, −25.1	−29.5, 0.2	−32.4, −1.1	−58.9, −28.6
P-value[a]		0.1303	**0.0036**	**<0.0001**	0.0526	**0.0358**	**<0.0001**

LS mean and LS mean differences are provided for categories with more than one patient in each treatment group. Baseline defined as the last nonmissing assessment, including any unscheduled or repeat assessments, collected prior to the first administration of study drug. Bold P-values denote those that are statistically significant (P<0.05). [a]Analysis performed using an analysis of covariance model with treatment group as a fixed effect and baseline value as the covariate. [b]Difference calculated as LS mean in active treatment minus placebo. CI, confidence interval; LS, least squares; MRI-PDFF, magnetic resonance imaging proton density fat fraction; SD, standard deviation; SE, standard error.

Supplementary Table 2 | Change from baseline in cT1 at Week 12 for all treatment groups (Efficacy Analysis Set)

	Placebo (*N*=24)	TERN-501 1 mg (*N*=23)	TERN-501 3 mg (*N*=23)	TERN-501 6 mg (*N*=22)	TERN-101 10 mg (*N*=24)	TERN-501 3 mg + TERN-101 10 mg (*N*=23)	TERN-501 6 mg + TERN-101 10 mg (*N*=23)
**Change (msec) from baseline at Week 12**							
n	21	22	19	18	23	18	21
LS mean (SE)[a]	3.6 (14.9)	−28.2 (14.6)	−25.6 (15.7)	−72.0 (16.1)	−35.4 (14.4)	−59.0 (16.3)	−65.5 (15.0)
LS mean difference (SE)[a,b]		−31.9 (20.9)	−29.3 (21.7)	−75.7 (22.0)	−39.0 (20.8)	−62.6 (22.1)	−69.2 (21.1)
95% CI for LS mean difference[a,b]		−73.2, 9.4	−72.1, 13.6	−119.1, −32.2	−80.1, 2.1	−106.3, −19.0	−111.0, −27.4
P-value[a]		0.1289	0.1790	**0.0008**	0.0628	**0.0053**	**0.0014**

LS mean and LS mean differences are provided for treatment groups with more than one patient. Baseline defined as the last nonmissing assessment, including any unscheduled or repeat assessments, collected prior to the first administration of study drug. Bold P-values denote those that are statistically significant (P<0.05). [a]Analysis performed using an analysis of covariance model with treatment group as a fixed effect and baseline value as the covariate. [b]Difference calculated as LS mean in active treatment minus placebo. CI, confidence interval; cT1, corrected T1; LS, least squares; SE, standard error.

Supplementary Table 3 | Percentage change from baseline in PD markers at Week 12 for all treatment groups (Efficacy Analysis Set)

	**Statistic**	**Placebo (*N*=24)**	**TERN-501 1 mg (*N*=23)**	**TERN-501 3 mg (*N*=23)**	**TERN-501 6 mg (*N*=22)**	**TERN-101 10 mg (*N*=24)**	**TERN-501 3 mg + TERN-101 10 mg (*N*=23)**	**TERN-501 6 mg + TERN-101 10 mg (*N*=23)**
SHBG	**Baseline**							
	n	22	22	22	22	23	23	23
	Mean (nmol/L) (SD)	46.2 (37.1)	40.6 (20.0)	39.7 (22.7)	32.3 (19.3)	39.1 (30.2)	36.6 (23.7)	35.1 (17.9)
	**Week 12**							
	n	20	23	20	21	23	18	20
	Mean (nmol/L) (SD)	38.5 (22.3)	44.4 (18.3)	64.5 (40.8)	66.7 (36.9)	45.8 (52.3)	64.3 (57.5)	75.6 (60.6)
	**Relative change from baseline**							
	LS mean (%) (SE)[a]	4.4 (17.3)	16.5 (15.7)	53.1 (16.9)	127.4 (16.1)	9.9 (15.7)	72.0 (17.3)	108.7 (16.5)
	LS mean difference (SE)[a,b]		12.2 (23.4)	48.8 (24.2)	123.0 (23.6)	5.6 (23.4)	67.6 (24.5)	104.3 (23.9)
	95% CI for LS mean difference[a,b]		−34.1, 58.4	0.9, 96.6	76.2, 169.7	−40.6, 51.8	19.2, 116.1	57.1, 151.6
	P-value		0.6039	**0.0458**	**<0.0001**	0.8117	**0.0066**	**<0.0001**
ApoB	**Baseline**							
	n	23	22	23	22	21	20	22
	Mean (mg/dL) (SD)	90.7 (19.3)	91.7 (18.3)	98.5 (19.4)	93.5 (19.2)	85.6 (19.1)	91.1 (24.1)	92.3 (17.0)
	**Week 12**							
	n	21	22	20	20	22	17	19
	Mean (mg/dL) (SD)	96.5 (20.8)	89.0 (19.2)	88.0 (15.8)	87.2 (21.9)	89.4 (21.9)	89.5 (19.5)	86.6 (17.5)
	**Relative change from baseline**							
	LS mean (%) (SE)[a]	4.9 (3.2)	−4.5 (3.1)	−8.1 (3.2)	−5.8 (3.2)	2.0 (3.2)	−4.2 (3.6)	−6.1 (3.4)
	LS mean difference (SE)[a,b]		−9.4 (4.5)	−13.0 (4.6)	−10.7 (4.5)	−2.9 (4.6)	−9.1 (4.8)	−11.0 (4.7)
	95% CI for LS mean difference[a,b]		−18.2, −0.5	−22.0, −4.0	−19.6, −1.7	−11.9, 6.1	−18.6, 0.5	−20.2, −1.7
	P-value		**0.0389**	**0.0051**	**0.0206**	0.5243	**0.0621**	**0.0204**
LDL-C	**Baseline**							
	n	23	22	23	22	21	20	22
	Mean (mg/dL) (SD)	87.3 (29.4)	101.6 (31.9)	101.7 (26.6)	98.8 (29.9)	84.9 (27.4)	89.4 (30.1)	93.0 (24.0)
	**Week 12**							
	n	21	22	20	20	22	17	19

	Statistic	Placebo (*N*=24)	TERN-501 1 mg (*N*=23)	TERN-501 3 mg (*N*=23)	TERN-501 6 mg (*N*=22)	TERN-101 10 mg (*N*=24)	TERN-501 3 mg + TERN-101 10 mg (*N*=23)	TERN-501 6 mg + TERN-101 10 mg (*N*=23)
	Mean (mg/dL) (SD)	93.1 (29.4)	92.5 (28.2)	88.9 (26.0)	90.3 (35.6)	93.0 (32.4)	87.5 (23.9)	87.6 (23.1)
	**Relative change from baseline**							
	LS mean (%) (SE)[a]	2.9 (5.2)	−3.6 (5.1)	−9.1 (5.2)	−7.7 (5.2)	6.8 (5.2)	−2.9 (5.8)	−4.1 (5.4)
	LS mean difference (SE)[a,b]		−6.4 (7.3)	−11.9 (7.3)	−10.6 (7.3)	3.9 (7.3)	−5.7 (7.7)	−6.9 (7.5)
	95% CI for LS mean difference[a,b]		−20.8, 7.9	−26.4, 2.6	−25.1, 4.0	−10.6, 18.4	−21.0, 9.6	-21.8, 7.9
	P-value		0.3759	0.1060	0.1526	0.5941	0.4626	0.3583
HDL-C	**Baseline**							
	n	23	22	23	22	21	20	22
	Mean (mg/dL) (SD)	43.1 (10.3)	47.6 (13.0)	46.4 (11.7)	42.8 (9.4)	39.9 (9.5)	42.6 (10.1)	43.0 (8.4)
	**Week 12**							
	n	21	22	20	20	22	17	19
	Mean (mg/dL) (SD)	44.5 (10.5)	46.0 (12.7)	44.9 (11.6)	46.5 (11.8)	39.4 (9.9)	38.6 (5.6)	38.7 (9.5)
	**Relative change from baseline**							
	LS mean (%) (SE)[a]	2.0 (3.1)	0.7 (3.1)	−1.0 (3.1)	9.3 (3.1)	1.2 (3.1)	−5.6 (3.5)	−9.7 (3.3)
	LS mean difference (SE)[a,b]		−1.3 (4.3)	−2.9 (4.4)	7.34 (4.4)	−0.8 (4.4)	−7.5 (4.6)	−11.7 (4.5)
	95% CI for LS mean difference[a,b]		−9.9, 7.3	−11.6, 5.7	−1.3, 16.0	−9.5, 7.9	−16.7, 1.7	−20.6, −2.8
	P-value		0.7710	0.5046	0.0958	0.8581	0.1071	**0.0104**
VLDL-C	**Baseline**							
	n	23	22	23	22	21	20	22
	Mean (mg/dL) (SD)	30.1 (13.2)	22.8 (9.3)	29.0 (12.2)	27.9 (12.0)	24.3 (7.4)	29.3 (21.1)	27.8 (10.9)
	**Week 12**							
	n	21	22	20	20	22	17	19
	Mean (mg/dL) (SD)	31.3 (18.6)	25.3 (18.9)	26.5 (10.1)	23.9 (10.8)	23.4 (7.9)	24.1 (15.0)	24.9 (7.7)
	**Relative change from baseline**							
	LS mean (%) (SE)[a]	8.0 (6.2)	−5.2 (6.1)	−1.6 (6.2)	−10.8 (6.2)	−7.3 (6.2)	−16.8 (6.9)	−5.7 (6.5)
	LS mean difference (SE)[a,b]		−13.1 (8.7)	−9.5 (8.7)	−18.8 (8.7)	−15.2 (8.8)	−24.7 (9.3)	−13.6 (9.0)
	95% CI for LS mean difference[a,b]		−30.4, 4.1	−26.8, 7.7	−36.1, −1.6	−32.6, 2.1	−43.0, −6.4	−31.4, 4.1
	P-value		0.1339	0.2758	**0.0329**	0.0848	**0.0085**	0.1313

	Statistic	Placebo (*N*=24)	TERN-501 1 mg (*N*=23)	TERN-501 3 mg (*N*=23)	TERN-501 6 mg (*N*=22)	TERN-101 10 mg (*N*=24)	TERN-501 3 mg + TERN-101 10 mg (*N*=23)	TERN-501 6 mg + TERN-101 10 mg (*N*=23)
TC	**Baseline**							
	n	23	22	23	22	21	20	22
	Mean (mg/dL) (SD)	160.5 (33.2)	172.0 (35.2)	177.1 (33.4)	169.5 (37.4)	149.1 (29.7)	161.2 (42.5)	163.8 (27.1)
	**Week 12**							
	n	21	22	20	20	22	17	19
	Mean (mg/dL) (SD)	169.0 (35.1)	163.8 (33.0)	160.3 (28.8)	160.7 (39.5)	155.8 (36.8)	150.3 (32.3)	151.3 (27.0)
	**Relative change from baseline**							
	LS mean (%) (SE)[a]	3.2 (3.2)	−3.8 (3.1)	−6.5 (3.2)	−4.1 (3.2)	1.8 (3.3)	−8.1 (3.6)	−7.1 (3.4)
	LS mean difference (SE)[a,b]		−7.0 (4.5)	−9.7 (4.6)	−7.3 (4.6)	−1.5 (4.6)	−11.3 (4.8)	−10.3 (4.7)
	95% CI for LS mean difference[a,b]		−16.0, 1.9	−18.8, −0.7	−16.3, 1.7	−10.5, 7.6	−20.8, −1.7	−19.6, −1.1
	P-value		0.1204	**0.0354**	0.1105	0.7501	**0.0210**	**0.0288**
TG	**Baseline**							
	n	23	22	23	22	21	20	22
	Mean (mg/dL) (SD)	176.3 (86.1)	127.9 (57.5)	165.1 (72.9)	156.9 (72.9)	136.6 (47.7)	163.8 (120.7)	158.4 (69.0)
	**Week 12**							
	n	21	22	20	20	22	17	19
	Mean (mg/dL) (SD)	183.6 (122.6)	144.1 (123.6)	152.8 (63.6)	133.4 (68.2)	129.0 (52.4)	132.5 (89.6)	140.2 (49.8)
	**Relative change from baseline**							
	LS mean (%) (SE)[a]	8.7 (7.0)	−5.9 (6.9)	0.2 (7.0)	−11.3 (7.0)	−9.3 (7.0)	−19.8 (7.9)	−6.5 (7.4)
	LS mean difference (SE)[a,b]		−14.5 (9.9)	−8.5 (9.9)	−19.9 (9.9)	−18.0 (10.0)	−28.4 (10.5)	−15.2 (10.2)
	95% CI for LS mean difference[a,b]		−34.2, 5.1	−28.1, 11.2	−39.6, −0.3	−37.7, 1.8	−49.3, −7.6	−35.4, 5.0
	P-value		0.1453	0.3959	**0.0470**	0.0746	**0.0078**	0.1392
Lp(a)	**Baseline**							
	n	22	23	22	22	22	23	22
	Mean (mg/dL) (SD)	13.6 (17.4)	22.6 (31.6)	24.4 (37.9)	37.6 (36.0)	14.8 (15.4)	16.4 (15.7)	30.0 (36.1)
	**Week 12**							
	n	20	22	19	21	22	19	21
	Mean (mg/dL) (SD)	14.5 (18.2)	21.2 (30.8)	25.4 (41.5)	33.2 (34.7)	15.6 (29.0)	12.7 (15.0)	15.2 (16.1)
	**Relative change from baseline**							

	Statistic	Placebo (*N*=24)	TERN-501 1 mg (*N*=23)	TERN-501 3 mg (*N*=23)	TERN-501 6 mg (*N*=22)	TERN-101 10 mg (*N*=24)	TERN-501 3 mg + TERN-101 10 mg (*N*=23)	TERN-501 6 mg + TERN-101 10 mg (*N*=23)
	LS mean (%) (SE)[a]	3.5 (5.3)	−9.2 (4.9)	−7.0 (5.4)	−16.9 (5.1)	−13.5 (5.2)	−15.6 (5.3)	−32.5 (5.0)
	LS mean difference (SE)[a,b]		−12.7 (7.2)	−10.5 (7.6)	−20.4 (7.5)	−17.0 (7.4)	−19.1 (7.5)	−36.0 (7.4)
	95% CI for LS mean difference[a,b]		−27.0, 1.6	−25.6, 4.5	−35.2, −5.6	−31.6, −2.4	−33.9, −4.3	−50.6, −21.4
	P-value		0.0821	0.1691	**0.0071**	**0.0226**	**0.0118**	**<0.0001**

Baseline is defined as the last nonmissing assessment, including any unscheduled or repeat assessments, collected prior to the first administration of study drug. Bold P-values denote those that are statistically significant (P<0.05). [a]Analysis is performed using an analysis of covariance model with treatment group as a fixed effect and baseline value as the covariate. [b]Difference is calculated as LS mean of treatment minus placebo. ApoB, apolipoprotein B; CI, confidence interval; HDL-C, high-density lipoprotein cholesterol; LDL-C, low-density lipoprotein cholesterol; Lp(a), lipoprotein(a); LS, least squares; PD, pharmacodynamic; SD, standard deviation; SE, standard error; SHBG, sex hormone-binding globulin; TC, total cholesterol; TG, triglyceride; VLDL-C, very low-density lipoprotein cholesterol.

Supplementary Table 4 | TEAEs reported in ≥5% of patients in any treatment group by Preferred Term (Safety Analysis Set)

**Preferred Term, *n* (%)**	**Placebo (*N*=24)**	**TERN-501 1 mg (*N*=23)**	**TERN-501 3 mg (*N*=23)**	**TERN-501 6 mg (*N*=22)**	**TERN-101 10 mg (*N*=24)**	**TERN-501 3 mg + TERN-101 10 mg (*N*=23)**	**TERN-501 6 mg + TERN-101 10 mg (*N*=23)**	**Total (*N*=162)**
Any TEAE	11 (45.8)	11 (47.8)	13 (56.5)	11 (50.0)	10 (41.7)	14 (60.9)	12 (52.2)	82 (50.6)
Pruritus	3 (12.5)	0	2 (8.7)	3 (13.6)	1 (4.2)	7 (30.4)	4 (17.4)	20 (12.3)
Diarrhea	1 (4.2)	1 (4.3)	5 (21.7)	2 (9.1)	1 (4.2)	2 (8.7)	0	12 (7.4)
Headache	2 (8.3)	1 (4.3)	1 (4.3)	1 (4.5)	1 (4.2)	2 (8.7)	0	8 (4.9)
Upper respiratory tract infection	0	1 (4.3)	0	1 (4.5)	2 (8.3)	3 (13.0)	0	7 (4.3)
Nausea	0	0	2 (8.7)	1 (4.5)	0	1 (4.3)	2 (8.7)	6 (3.7)
Nasopharyngitis	0	0	1 (4.3)	2 (9.1)	1 (4.2)	0	0	4 (2.5)

A TEAE is any adverse event with a start date on or after the date of first administration of study drug through 30 days after the last administration of study drug or through the follow-up period (Week 16). Patients are counted once for each Preferred Term. TEAE, treatment-emergent adverse event.

Supplementary Table 5 | Percentage change from baseline in sex hormones at Week 12 for all treatment groups (Safety Analysis Set)

		Statistic	Placebo (*N*=24)	TERN-501 1 mg (*N*=23)	TERN-501 3 mg (*N*=23)	TERN-501 6 mg (*N*=22)	TERN-101 10 mg (*N*=24)	TERN-501 3 mg + TERN-101 10 mg (*N*=23)	TERN-501 6 mg + TERN-101 10 mg (*N*=23)
Free testosterone	Male	**Baseline**							
		n	7	8	5	4	7	8	9
		Mean (pg/mL) (SD)	39.4 (13.2)	37.5 (14.0)	36.3 (11.9)	37.3 (17.2)	48.3 (36.7)	30.9 (12.3)	29.7 (8.5)
		**Week 12**							
		n	5	9	5	4	7	6	7
		Mean (pg/mL) (SD)	32.6 (12.2)	32.6 (12.4)	34.8 (15.7)	45.4 (17.3)	56.0 (39.0)	25.2 (14.1)	48.1 (55.7)
		**Relative change from baseline**							
		LS mean (%) (SE)[a]	−6.5 (3.4)	−6.1 (2.9)	13.5 (5.4)	0.4 (5.5)	−0.4 (3.2)	−4.7 (3.5)	−5.2 (3.5)
		LS mean difference (SE)[a,b]		0.4 (4.5)	20.0 (6.4)	6.9 (6.4)	6.1 (4.7)	1.8 (4.9)	1.3 (4.9)
		95% CI for LS mean difference[a,b]		−8.8, 9.7	6.7, 33.3	−6.4, 20.2	−3.6, 15.7	−8.3, 11.9	−8.8, 11.4
		P-value		0.9216	**0.0048**	0.2955	0.2084	0.7183	0.7912
	Female	**Baseline**							
		n	13	11	12	16	12	11	10
		Mean (pg/mL) (SD)	4.1 (3.4)	4.3 (2.1)	8.3 (4.8)	5.6 (4.3)	6.1 (6.7)	3.7 (2.0)	4.1 (2.2)
		**Week 12**							
		n	12	11	11	15	11	9	8
		Mean (pg/mL) (SD)	5.4 (4.5)	4.9 (2.4)	7.6 (4.9)	6.2 (4.3)	6.1 (7.7)	3.5 (1.9)	3.7 (2.1)
		**Relative change from baseline**							
		LS mean (%) (SE)[a]	0.2 (0.6)	0.5 (0.6)	−0.5 (0.6)	0.7 (0.5)	0.3 (0.6)	0.0 (0.7)	−0.8 (0.7)
		LS mean difference (SE)[a,b]		0.3 (0.8)	−0.7 (0.9)	0.5 (0.8)	0.0 (0.8)	−0.3 (0.9)	−1.0 (0.9)
		95% CI for LS mean difference[a,b]		−1.4, 2.0	−2.5, 1.1	−1.1, 2.0	−1.6, 1.7	−2.0, 1.5	−2.9, 0.8
		P-value		0.7047	0.4304	0.5648	0.9542	0.7765	0.2653

		Statistic	Placebo (*N*=24)	TERN-501 1 mg (*N*=23)	TERN-501 3 mg (*N*=23)	TERN-501 6 mg (*N*=22)	TERN-101 10 mg (*N*=24)	TERN-501 3 mg + TERN-101 10 mg (*N*=23)	TERN-501 6 mg + TERN-101 10 mg (*N*=23)
Estradiol	Male	**Baseline**							
		n	9	11	10	6	11	12	11
		Mean (pg/mL) (SD)	28.1 (13.1)	31.3 (11.6)	24.7 (6.3)	26.9 (7.0)	37.2 (16.5)	28.0 (10.6)	25.0 (6.6)
		**Week 12**							
		n	9	12	9	6	12	10	10
		Mean (pg/mL) (SD)	22.3 (6.2)	30.7 (11.1)	29.3 (16.6)	34.4 (12.4)	35.8 (12.2)	32.1 (14.1)	29.8 (8.5)
		**Relative change from baseline**							
		LS mean (%) (SE)[a]	−6.3 (4.0)	2.4 (3.6)	1.6 (4.1)	6.0 (4.9)	6.2 (3.8)	3.5 (3.8)	1.8 (3.8)
		LS mean difference (SE)[a,b]		8.7 (5.4)	7.9 (5.7)	12.3 (6.3)	12.5 (5.5)	9.8 (5.5)	8.2 (5.5)
		95% CI for LS mean difference[a,b]		−2.1, 19.5	−3.5, 19.3	−0.3, 24.9	1.5, 23.5	−1.1, 20.8	−2.9, 19.2
		P-value		0.1119	0.1687	0.0553	**0.0272**	0.0781	0.1437
	Female	**Baseline**							
		n	14	11	13	15	12	11	11
		Mean (pg/mL) (SD)	35.7 (43.6)	36.3 (45.8)	39.1 (36.2)	33.4 (25.7)	28.8 (15.6)	31.9 (21.0)	44.3 (46.5)
		**Week 12**							
		n	12	11	11	15	11	9	8
		Mean (pg/mL) (SD)	31.4 (32.7)	48.2 (51.0)	40.0 (61.2)	36.5 (40.1)	31.7 (23.5)	47.1 (54.3)	62.3 (73.0)
		**Relative change from baseline**							
		LS mean (%) (SE)[a]	3.6 (9.6)	11.7 (9.5)	0.3 (9.5)	3.0 (8.4)	5.8 (9.5)	14.7 (10.5)	9.0 (11.3)
		LS mean difference (SE)[a,b]		8.1 (13.5)	−3.3 (13.6)	−0.6 (12.8)	2.3 (13.4)	11.2 (14.2)	5.4 (15.1)
		95% CI for LS mean difference[a,b]		−18.9, 35.1	−30.4, 23.8	−26.1, 24.9	−24.5, 29.0	−17.2, 39.5	−24.7, 35.4
		P-value		0.5511	0.8095	0.9628	0.8666	0.4336	0.7213

		Statistic	Placebo (*N*=24)	TERN-501 1 mg (*N*=23)	TERN-501 3 mg (*N*=23)	TERN-501 6 mg (*N*=22)	TERN-101 10 mg (*N*=24)	TERN-501 3 mg + TERN-101 10 mg (*N*=23)	TERN-501 6 mg + TERN-101 10 mg (*N*=23)
FSH	Male	**Baseline**							
		n	4	5	5	2	4	6	6
		Mean (mIU/mL) (SD)	3.9 (0.8)	11.6 (15.3)	8.0 (4.6)	4.2 (3.4)	6.3 (4.9)	4.3 (2.9)	6.6 (4.2)
		**Week 12**							
		n	3	5	5	5	3	7	4
		Mean (mIU/mL) (SD)	3.1 (0.4)	10.7 (15.3)	6.0 (3.6)	6.5 (2.8)	6.6 (6.8)	5.2 (3.7)	6.4 (4.7)
		**Relative change from baseline**							
		LS mean (%) (SE)[a]	−0.7 (0.7)	−0.8 (0.5)	−1.1 (0.7)	0.9 (0.8)	−0.2 (0.7)	0.0 (0.5)	−1.2 (0.6)
		LS mean difference (SE)[a,b]		−0.1 (0.9)	−0.5 (1.0)	1.5 (1.0)	0.4 (0.9)	0.6 (0.8)	−0.6 (0.9)
		95% CI for LS mean difference[a,b]		−2.0, 1.7	−2.5, 1.5	−0.7, 3.7	−1.5, 2.4	−1.1, 2.4	−2.4, 1.3
		P-value		0.8722	0.6275	0.1644	0.6411	0.4593	0.5314
	Female	**Baseline**							
		n	14	7	10	16	12	11	9
		Mean (mIU/mL) (SD)	40.0 (30.8)	17.9 (15.1)	29.8 (31.0)	33.3 (22.2)	32.7 (27.3)	28.4 (18.8)	35.3 (21.1)
		**Week 12**							
		n	12	7	9	14	11	7	8
		Mean (mIU/mL) (SD)	41.6 (25.2)	26.1 (28.7)	32.8 (25.1)	33.0 (20.4)	29.0 (20.0)	24.7 (13.6)	32.9 (23.0)
		**Relative change from baseline**							
		LS mean (%) (SE)[a]	−5.6 (3.0)	−5.2 (4.0)	1.9 (3.4)	−2.2 (2.6)	−4.8 (2.9)	−6.8 (3.6)	−0.6 (3.6)
		LS mean difference (SE)[a,b]		0.4 (5.1)	7.5 (4.5)	3.4 (3.9)	0.8 (4.2)	−1.2 (4.7)	5.0 (4.7)
		95% CI for LS mean difference[a,b]		−9.8, 10.6	−1.6, 16.6	−4.4, 11.3	−7.5, 9.1	−10.7, 8.2	−4.4, 14.4
		P-value		0.9416	0.1024	0.3871	0.8498	0.7982	0.2875

		Statistic	Placebo (*N*=24)	TERN-501 1 mg (*N*=23)	TERN-501 3 mg (*N*=23)	TERN-501 6 mg (*N*=22)	TERN-101 10 mg (*N*=24)	TERN-501 3 mg + TERN-101 10 mg (*N*=23)	TERN-501 6 mg + TERN-101 10 mg (*N*=23)
LH	Male	**Baseline**							
		n	9	11	10	6	11	12	12
		Mean (mIU/mL) (SD)	3.4 (1.2)	4.5 (3.2)	3.9 (2.0)	4.4 (1.6)	4.5 (3.2)	4.3 (4.0)	4.0 (2.0)
		**Week 12**							
		n	9	12	9	6	12	10	10
		Mean (mIU/mL) (SD)	3.3 (1.0)	4.8 (3.9)	4.9 (2.2)	4.8 (1.7)	4.3 (3.5)	5.7 (7.3)	4.8 (2.8)
		**Relative change from baseline**							
		LS mean (%) (SE)[a]	0.0 (0.7)	0.4 (0.6)	1.0 (0.7)	0.4 (0.9)	−0.3 (0.6)	1.3 (0.7)	0.4 (0.7)
		LS mean difference (SE)[a,b]		0.4 (1.0)	1.0 (1.0)	0.4 (1.1)	−0.3 (1.0)	1.3 (1.0)	0.4 (1.0)
		95% CI for LS mean difference[a,b]		−1.6, 2.3	−1.0, 3.0	−1.9, 2.6	−2.2, 1.6	−0.7, 3.3	−1.5, 2.4
		P-value		0.6933	0.3134	0.7353	0.7472	0.1937	0.6575
	Female	**Baseline**							
		n	15	11	13	16	12	11	11
		Mean (mIU/mL) (SD)	20.2 (14.4)	21.3 (14.9)	19.0 (12.8)	20.2 (13.8)	16.0 (9.2)	10.8 (7.6)	20.0 (10.6)
		**Week 12**							
		n	12	11	11	15	11	9	9
		Mean (mIU/mL) (SD)	21.0 (12.7)	18.7 (13.1)	22.6 (12.1)	21.1 (14.2)	15.8 (9.6)	13.8 (8.5)	20.5 (9.3)
		**Relative change from baseline**							
		LS mean (%) (SE)[a]	−2.8 (1.6)	−2.2 (1.6)	2.5 (1.6)	0.7 (1.4)	−0.4 (1.6)	1.3 (1.8)	2.4 (1.8)
		LS mean difference (SE)[a,b]		0.6 (2.2)	5.3 (2.3)	3.5 (2.1)	2.4 (2.3)	4.1 (2.5)	5.2 (2.4)
		95% CI for LS mean difference[a,b]		−3.9, 5.1	0.8, 9.8	−0.6, 7.7	−2.2, 6.9	−0.8, 9.0	0.5, 10.0
		P-value		0.7992	**0.0205**	0.0956	0.3056	0.0982	**0.0320**

Baseline is defined as the last nonmissing assessment, including any unscheduled or repeat assessments, collected prior to the first administration of study drug. Bold P-values denote those that are statistically significant (P<0.05). [a]Analysis is performed using an analysis of covariance model with treatment group as a fixed effect and baseline value as the covariate. [b]Difference is calculated as LS mean of treatment minus placebo. CI, confidence interval; LS, least squares; SD, standard deviation; SE, standard error; FSH, follicle-stimulating hormone; LH, luteinizing hormone.

Supplementary Table 6 | Glucose over time for all treatment groups (Safety Analysis Set)

	Statistic	Placebo (*N*=24)	TERN-501 1 mg (*N*=23)	TERN-501 3 mg (*N*=23)	TERN-501 6 mg (*N*=22)	TERN-101 10 mg (*N*=24)	TERN-501 3 mg + TERN-101 10 mg (*N*=23)	TERN-501 6 mg + TERN-101 10 mg (*N*=23)
Glucose	**Baseline**							
	n	24	23	23	22	24	23	23
	Mean (mg/dL) (SD)	117.5 (36.10)	114.4 (26.76)	119.1 (37.46)	107.2 (27.16)	121.3 (42.51)	129.3 (50.56)	133.2 (43.19)
	**Week 2**							
	n	23	23	22	22	24	23	21
	Mean (mg/dL) (SD)	118.3 (38.99)	113.8 (23.85)	133.5 (68.41)	112.7 (33.56)	118.8 (28.19)	154.1 (66.70)	150.5 (51.71)
	**Week 4**							
	n	22	23	22	20	23	23	21
	Mean (mg/dL) (SD)	127.9 (34.49)	116.8 (27.06)	121.2 (41.40)	110.6 (28.15)	121.9 (37.56)	141.4 (49.56)	176.0 (82.02)
	**Week 6**							
	n	20	23	21	20	22	20	22
	Mean (mg/dL) (SD)	122.6 (29.75)	116.2 (29.44)	130.3 (56.81)	114.8 (26.08)	113.6 (33.66)	151.2 (74.04)	149.8 (52.81)
	**Week 8**							
	n	21	23	22	19	23	18	21
	Mean (mg/dL) (SD)	121.9 (31.81)	114.2 (31.44)	127.6 (51.82)	112.8 (23.72)	117.4 (37.87)	143.4 (50.41)	162.2 (58.92)
	**Week 12**							
	n	20	22	19	21	22	19	21
	Mean (mg/dL) (SD)	129.7 (51.89)	120.9 (53.01)	138.6 (90.85)	108.3 (23.94)	109.9 (32.25)	149.9 (63.80)	151.1 (71.34)
	**Week 16**							
	n	21	23	20	22	22	19	20
	Mean (mg/dL) (SD)	122.0 (34.52)	129.6 (58.79)	130.7 (40.17)	114.5 (29.90)	113.5 (30.28)	147.3 (63.47)	127.7 (42.57)

Supplementary Table 7 | Change from baseline in exploratory biomarkers at Week 12 (Efficacy Analysis Set)

	**Statistic**	**Placebo (*N*=24)**	**TERN-501 1 mg (*N*=23)**	**TERN-501 3 mg (*N*=23)**	**TERN-501 6 mg (*N*=22)**	**TERN-101 10 mg (*N*=24)**	**TERN-501 3 mg + TERN-101 10 mg (*N*=23)**	**TERN-501 6 mg + TERN-101 10 mg (*N*=23)**
ALT	*n*	20	22	19	21	22	19	20
	Mean (U/L) (SD)	44.4 (17.77)	43.5 (29.83)	35.5 (13.80)	35.2 (18.13)	41.6 (23.74)	46.8 (34.95)	38.1 (21.38)
	LS mean change from baseline (SE)[a]	1.0 (4.25)	0.8 (4.05)	−5.6 (4.36)	−3.6 (4.16)	1.0 (4.05)	3.7 (4.36)	−7.2 (4.26)
	P-value[a]		0.9822	0.2825	0.4481	0.9972	0.6574	0.1773
AST	*n*	20	22	19	21	22	19	21
	Mean (U/L) (SD)	33.9 (13.23)	32.6 (22.69)	28.4 (10.19)	29.0 (16.05)	33.5 (15.04)	35.1 (22.92)	29.1 (12.20)
	LS mean change from baseline (SE)[a]	1.1 (3.03)	1.8 (2.88)	−1.6 (3.10)	2.1 (2.98)	2.5 (2.88)	4.5 (3.10)	−2.6 (2.95)
	P-value[a]		0.8617	0.5417	0.8105	0.7321	0.4342	0.3849
GGT	*n*	20	22	19	21	22	19	21
	Mean (U/L) (SD)	46.6 (22.57)	37.6 (23.55)	40.4 (19.59)	34.7 (20.87)	36.9 (25.86)	33.7 (23.70)	28.7 (12.34)
	LS mean change from baseline (SE)[a]	−2.8 (3.14)	−3.7 (2.98)	−2.3 (3.20)	−3.1 (3.08)	−11.2 (2.98)	−13.2 (3.20)	−17.3 (3.05)
	P-value[a]		0.8277	0.9195	0.9455	0.0522	**0.0216**	**0.0011**
TE	*n*	21	23	20	20	23	19	21
	Mean (kPa) (SD)	9.9 (5.5)	9.3 (3.9)	8.9 (6.5)	8.8 (3.9)	8.7 (3.3)	9.5 (4.1)	9.4 (3.2)
	LS mean change from baseline (SE)[a]	−0.55 (0.8)	−1.49 (0.8)	−0.99 (0.8)	−1.21 (0.8)	−2.11 (0.8)	−0.80 (0.9)	−2.03 (0.8)
	P-value[a]		0.4081	0.7091	0.5751	0.1677	0.8330	0.2046
CAP	*n*	21	23	20	20	23	19	21
	Mean (dB/m) (SD)	331.6 (35.8)	335.2 (44.5)	297.9 (58.6)	297.0 (45.2)	314.9 (34.3)	322.7 (50.7)	320.7 (41.4)
	LS mean change from baseline (SE)[a]	−8.8 (9.1)	1.5 (8.8)	−43.7 (9.4)	−45.6 (9.4)	−23.3 (8.7)	−15.7 (9.6)	−20.0 (9.2)
	P-value[a]		0.4219	**0.0086**	**0.0057**	0.2544	0.6034	0.3873
FAST score	*n*	20	22	19	21	22	19	20
	Mean (SD)	0.41 (0.21)	0.36 (0.27)	0.30 (0.22)	0.28 (0.19)	0.35 (0.22)	0.35 (0.24)	0.33 (0.19)
	LS mean change from baseline (SE)[a]	−0.01 (0.04)	−0.02 (0.04)	−0.07 (0.04)	−0.07 (0.04)	−0.06 (0.04)	−0.04 (0.04)	−0.11 (0.04)
	P-value[a]		0.8771	0.2782	0.2978	0.4102	0.6045	0.0790

ELF score	*n*	21	22	20	21	23	19	20
	Mean (SD)	9.4 (0.8)	9.4 (0.8)	9.5 (0.9)	9.2 (0.6)	9.4 (0.6)	9.2 (0.7)	9.3 (0.6)
	LS mean change from baseline (SE)[a]	0.07 (0.10)	0.05 (0.10)[b]	0.14 (0.10)	0.19 (0.10)	−0.06 (0.10)[c]	−0.08 (0.11)	−0.05 (0.10)
	P-value[a]		0.9091	0.607	0.4077	0.3614	0.3273	0.4146
Hyaluronic acid	*n*	21	22	20	21	23	19	20
	Mean (ng/mL) (SD)	67.2 (43.5)	65.3 (58.1)	68.7 (52.1)	53.0 (42.8)	59.7 (39.6)	50.3 (39.8)	52.2 (29.8)
	LS mean change from baseline (SE)[a]	8.4 (7.7)	11.8 (7.7)[b]	8.1 (7.9)	8.5 (7.8)	−2.8 (7.5)[c]	−7.4 (8.1)	−6.0 (7.9)
	P-value[a]		0.7549	0.9848	0.9928	0.2995	0.1610	0.1955
PRO-C3	*n*	21	23	20	21	23	19	21
	Mean (ng/mL) (SD)	36.9 (6.6)	41.9 (11.6)	38.1 (10.8)	36.7 (6.7)	37.7 (9.1)	36.1 (7.1)	39.4 (11.7)
	LS mean change from baseline (SE)[a]	−0.4 (1.5)	2.3 (1.4)	0.2 (1.5)	0.6 (1.5)	0.2 (1.5)[c]	1.1 (1.6)	−0.4 (1.5)
	P-value[a]		0.1885	0.7731	0.6439	0.7720	0.4998	0.9803
CK-18 M30	*n*	21	23	20	21	22	19	20
	Mean (U/L) (SD)	393.3 (255.2)	356.1 (337.0)	271.0 (108.3)	270.6 (135.6)	479.6 (596.2)	332.2 (230.5)	312.7 (184.3)
	LS mean change from baseline (SE)[a]	15.3 (48.4)[d]	51.0 (45.1)	−29.4 (48.4)	8.5 (47.5)	74.7 (47.7)[b]	20.4 (49.6)	−39.0 (48.3)
	P-value[a]		0.5918	0.5158	0.9201	0.3807	0.9415	0.4283
CK-18 M65	*n*	21	23	20	21	22	19	20
	Mean (U/L) (SD)	601.4 (369.7)	486.6 (479.9)	386.6 (138.3)	381.9 (227.8)	429.7 (280.4)	534.1 (388.1)	401.7 (255.8)
	LS mean change from baseline (SE)[a]	56.0 (62.7)[d]	36.5 (57.8)	−72.2 (62.0)	−35.9 (60.9)	−30.7 (60.4)[b]	40.5 (63.6)	−94.7 (62.0)
	P-value[a]		0.8198	0.1493	0.2991	0.3212	0.8616	0.0884
rT3	*n*	19	22	19	20	21	15	21
	Mean (ng/dL) (SD)	19.6 (4.3)	18.2 (5.0)	16.4 (7.3)	15.7 (7.2)	18.3 (4.5)	16.6 (7.1)	15.6 (4.8)
	LS mean change from baseline (SE)[a]	0.4 (1.3)[e]	−0.7 (1.1)[b]	−0.7 (1.3)[e]	−4.0 (1.2)[f]	−0.1 (1.3)[e]	−2.3 (1.3)	−3.0 (1.1)
	P-value[a]		0.4884	0.5254	**0.0111**	0.7542	0.1337	**0.0442**
fT3/rT3 ratio	*n*	19	22	19	20	21	15	21
	Mean (SD)	18.2 (5.1)	21.6 (8.6)	25.9 (10.3)	26.6 (15.9)	20.6 (4.6)	22.2 (7.6)	24.1 (9.5)
	LS mean change from baseline (SE)[a]	−1.0 (2.2)[e]	2.1 (1.9)[b]	2.6 (2.2)[e]	8.2 (2.1)[f]	−0.3 (2.2)[e]	2.6 (2.3)	4.6 (2.0)
	P-value[a]		0.2953	0.2603	**0.0027**	0.8407	0.2565	0.0606

FIB-4	*n*	18	21	19	20	21	19	19
	Mean (SD)	0.3 (0.1)	0.3 (0.1)	0.4 (0.2)	0.3 (0.2)	0.4 (0.2)	0.3 (0.2)	0.3 (0.1)
	LS mean change from baseline (SE)[a]	0.02 (0.02)	0.02 (0.02)	0.03 (0.02)[g]	0.03 (0.02)	0.04 (0.02)	0.03 (0.02)	0.01 (0.02)
	P-value[a]		0.9246	0.6290	0.5913	0.4010	0.7098	0.8517
NFS	*n*	18	21	19	20	21	19	19
	Mean (SD)	−1.5 (1.1)	−1.3 (1.5)	−1.2 (1.6)	−1.7 (1.9)	−1.8 (1.4)	−1.0 (1.1)	−1.2 (1.2)
	LS mean change from baseline (SE)[a]	0.23 (0.15)	0.27 (0.14)	0.29 (0.15)[g]	0.12 (0.14)	0.09 (0.14)	0.10 (0.15)	0.03 (0.15)
	P-value[a]		0.831	0.7579	0.5943	0.5059	0.5503	0.3487
APRI	*n*	18	21	19	20	21	19	19
	Mean (SD)	0.33 (0.12)	0.35 (0.25)	0.29 (0.11)	0.26 (0.14)	0.31 (0.14)	0.33 (0.23)	0.30 (0.19)
	LS mean change from baseline (SE)[a]	0.04 (0.04)[h]	0.04 (0.03)[d]	−0.01 (0.04)[h]	0.02 (0.03)	0.01 (0.04)[i]	0.03 (0.04)[e]	−0.02 (0.03)
	P-value[a]		0.9677	0.3789	0.7180	0.6646	0.8851	0.2647

[a]Analysis performed using a type III sum of squares analysis of covariance model with treatment group as a fixed effect and baseline value as the covariate. A hierarchical testing strategy was employed. Bold P-values denote those that are statistically significant (P<0.05). [b]*n*=21. [c]*n*=22. [d]*n*=20. [e]*n*=17. [f]*n*=19. [g]*n*=18. [h]*n*=15. [i]*n*=16. ALT, alanine transaminase; APRI, aspartate aminotransferase to platelet ratio; AST, aspartate aminotransferase; CAP, controlled attenuation parameter; CK-18 M30, cytokeratin 18 fragment M30; CK-18 M65, cytokeratin 18 fragment M65; ELF, enhanced liver fibrosis; FAST, FibroScan-aspartate aminotransferase; FIB-4, fibrosis-4; fT3, free triiodothyronine; GGT, gamma-glutamyl transferase; LS, least squares; NFS, Nonalcoholic fatty liver disease Fibrosis Score; PRO-C3, released N-terminal propeptide of type III collagen; rT3, reverse triiodothyronine; SD, standard deviation; SE, standard error; TE, transient elastography.
