# Supplementary material for: TERN-501 monotherapy and combination therapy with TERN-101 in metabolic dysfunction-associated steatohepatitis: the randomized phase 2a DUET trial
Source: Nat Med. 2025 Jun 11;31(7):2297–305. doi: 10.1038/s41591-025-03722-7 (PMC12283411; doi:10.1038/s41591-025-03722-7)
Supplement: Supplementary file 2 — Reporting Summary [file 41591_2025_3722_MOESM2_ESM.pdf]

nature portfolio

Corresponding author(s): Mazen Noureddin

Last updated by author(s): 07/04/2025

Reporting Summary

Nature Portfolio wishes to improve the reproducibility of the work that we publish. This form provides structure for consistency and transparency in reporting. For further information on Nature Portfolio policies, see our Editorial Policies and the Editorial Policy Checklist.

Statistics

For all statistical analyses, confirm that the following items are present in the figure legend, table legend, main text, or Methods section.

n/a	Confirmed	
☐	☒	The exact sample size (*n*) for each experimental group/condition, given as a discrete number and unit of measurement
☒	☐	A statement on whether measurements were taken from distinct samples or whether the same sample was measured repeatedly
☐	☒	The statistical test(s) used AND whether they are one- or two-sided<br>*Only common tests should be described solely by name; describe more complex techniques in the Methods section.*
☐	☒	A description of all covariates tested
☐	☒	A description of any assumptions or corrections, such as tests of normality and adjustment for multiple comparisons
☐	☒	A full description of the statistical parameters including central tendency (e.g. means) or other basic estimates (e.g. regression coefficient) AND variation (e.g. standard deviation) or associated estimates of uncertainty (e.g. confidence intervals)
☐	☒	For null hypothesis testing, the test statistic (e.g. *F*, *t*, *r*) with confidence intervals, effect sizes, degrees of freedom and *P* value noted<br>*Give P values as exact values whenever suitable.*
☒	☐	For Bayesian analysis, information on the choice of priors and Markov chain Monte Carlo settings
☒	☐	For hierarchical and complex designs, identification of the appropriate level for tests and full reporting of outcomes
☒	☐	Estimates of effect sizes (e.g. Cohen's *d*, Pearson's *r*), indicating how they were calculated

Our web collection on statistics for biologists contains articles on many of the points above.

Software and code

Policy information about availability of computer code

Data collection	NA
Data analysis	SAS® Version 9.4 for statistical/data analysis; noncompartmental PK parameters were estimated from individual plasma concentration data using Phoenix WinNonlin® Version 8.3.5.

For manuscripts utilizing custom algorithms or software that are central to the research but not yet described in published literature, software must be made available to editors and reviewers. We strongly encourage code deposition in a community repository (e.g. GitHub). See the Nature Portfolio guidelines for submitting code & software for further information.

Data

Policy information about availability of data

All manuscripts must include a data availability statement. This statement should provide the following information, where applicable:

- Accession codes, unique identifiers, or web links for publicly available datasets
- A description of any restrictions on data availability
- For clinical datasets or third party data, please ensure that the statement adheres to our policy

Datasets generated as part of the DUET study are considered sensitive and, as such, are not publicly available. Requests for data supporting the findings in this manuscript should be made to the corresponding author (M.N.: NoureddinMD@houstonresearchinstitute.com) and will be considered on a case-by-case basis 3 years after publication. Data may be shared in the form of aggregate data summaries and via a data transfer agreement with qualified noncommercial, scientific and

medical researchers at the researcher's request. Timescales vary depending on the request and may take several months after full submission of the request for sharing of the requested data or documents. Individual patient-level data are subject to patient privacy and cannot be shared.

Research involving human participants, their data, or biological material

Policy information about studies with human participants or human data. See also policy information about sex, gender (identity/presentation), and sexual orientation and race, ethnicity and racism.

Reporting on sex and gender	Biological sex has been reported in the manuscript. Findings apply to both sexes.<br>Sex was patient reported and based on medical records.<br>Data on number of male and female participants within each study arm have been provided in the article. Sex-based analyses were not performed as the study was not powered to do so, and currently there is no evidence to indicate a sex-specific difference in the pathology of MASH and mechanism of action of the study drug. For the same reason, sex and gender were not considered in the study design.
Reporting on race, ethnicity, or other socially relevant groupings	Demographics were patient reported and determined from medical records. No analyses were undertaken using socially constructed or socially relevant catergorization variables.
Population characteristics	All covariate-relevant population characteristics are reported in Table 1.
Recruitment	Most patients enrolled on DUET were already known to the investigators from patient databases. No recruitment vendors were used. Potential patients were identified on the basis of their diagnosis by the investigators or staff at study sites Patients to be enrolled was at the determination of the principal investigators. All screening failed along with reason(s) of screen failure were documented.
Ethics oversight	The clinical study protocol and its amendments, and the informed consent forms and their amendments, were reviewed and approved by an Institutional Review Board (IRB) or Independent Ethics Committee (IEC) at each study site, which are listed in the Supplementary Information. The Investigator was responsible for submitting the protocol, protocol amendments, ICF, Investigator Brochure, and other relevant documents (eg, advertisements) to an IRB/IEC. Written approval of these documents must have been obtained from the IRB/IEC before any patient was enrolled at the site.<br>Any amendments to the protocol required IRB/IEC approval before implementation of changes made to the study design, except for changes necessary to eliminate an immediate hazard to study patients.

Note that full information on the approval of the study protocol must also be provided in the manuscript.

Field-specific reporting

Please select the one below that is the best fit for your research. If you are not sure, read the appropriate sections before making your selection.

☒ Life sciences ☐ Behavioural & social sciences ☐ Ecological, evolutionary & environmental sciences

For a reference copy of the document with all sections, see nature.com/documents/nr-reporting-summary-flat.pdf

Life sciences study design

All studies must disclose on these points even when the disclosure is negative.

Sample size	The optimal sample size was determined as approximately 140 patients, randomly assigned to one of the seven study groups. Based on an assumed pooled SD of 22% with a two-sided alpha of 0.05, a mean relative reduction difference in MRI-PDFF at Week 12 of 23% between TERN-501 monotherapy and placebo would provide approximately 90% power, and a mean relative reduction difference in MRI-PDFF at Week 12 of 36% between TERN-501 plus TERN-101 combination therapy and placebo would provide over 90% power. Based on an assumed pooled SD of 82 msec with a two-sided alpha of 0.05, a mean reduction difference in cT1 at Week 12 of 77 msec between TERN 510 monotherapy and placebo would provide approximately 82% power, and a mean reduction difference in cT1 at Week 12 of 134 msec between TERN-501 plus TERN-101 combination therapy and placebo would provide over 90% power.
Data exclusions	12 patients were excluded from the per protocol analysis due to at least one important protocol deviation, including deviation in informed consent (n=1), study conduct/procedure (n=5), and deviations not defined in the protocol (other) (n=10)
Replication	This was a randomized controlled trial with human participants. Sample sizes were calculated to ensure the outcomes were powered for statistical significance without replication.
Randomization	Patients were randomized equally (1:1:1:1:1:1:1) into the 7 treatment groups using an Interactive Web Response System. The patient identification number was obtained after the ICF was signed, and the randomized treatment was assigned after patient eligibility was confirmed.
Blinding	Patients, investigators, and study personnel were masked to treatment assignment during the study.

Reporting for specific materials, systems and methods

We require information from authors about some types of materials, experimental systems and methods used in many studies. Here, indicate whether each material, system or method listed is relevant to your study. If you are not sure if a list item applies to your research, read the appropriate section before selecting a response.

Materials & experimental systems

n/a	Involved in the study
☒	☐ Antibodies
☒	☐ Eukaryotic cell lines
☒	☐ Palaeontology and archaeology
☒	☐ Animals and other organisms
☐	☒ Clinical data
☒	☐ Dual use research of concern
☒	☐ Plants

Methods

n/a	Involved in the study
☒	☐ ChIP-seq
☒	☐ Flow cytometry
☒	☐ MRI-based neuroimaging

Clinical data

Policy information about clinical studies

All manuscripts should comply with the ICMJE guidelines for publication of clinical research and a completed CONSORT checklist must be included with all submissions.

Clinical trial registration: NCT05415722

Study protocol: The study protocol contains commercially sensitive information and will not be made publicly available. This will be available upon request to the corresponding author.

Data collection: Data collection occurred between 17 June 2022 (first patient screened) and 10 July 2023 (last patient visit). The screening period duration was 9 months. Data, including laboratory tests and clinical assessments including MRIs, was collected at the patient's local study site, which are listed in full at https://clinicaltrials.gov/study/NCT05415722?tab=history&a=8#contacts-locations-card

Outcomes: The primary endpoint was the relative change from baseline in liver fat content, assessed by MRI-PDFF at Week 12, for TERN-501 versus placebo. Secondary endpoints included: change from baseline in liver fibroinflammation (assessed by cT1 relaxation time) at Week 12 for treatment groups receiving TERN-501, or TERN-501 in combination with TERN-101, versus placebo; and relative change from baseline in liver fat content assessed by MRI-PDFF at Week 12 for TERN-501 plus TERN-101 combination therapy groups versus placebo. Safety endpoints included the incidence of treatment-emergent adverse events.

Plants

Seed stocks: *Report on the source of all seed stocks or other plant material used. If applicable, state the seed stock centre and catalogue number. If plant specimens were collected from the field, describe the collection location, date and sampling procedures.*

Novel plant genotypes: *Describe the methods by which all novel plant genotypes were produced. This includes those generated by transgenic approaches, gene editing, chemical/radiation-based mutagenesis and hybridization. For transgenic lines, describe the transformation method, the number of independent lines analyzed and the generation upon which experiments were performed. For gene-edited lines, describe the editor used, the endogenous sequence targeted for editing, the targeting guide RNA sequence (if applicable) and how the editor was applied.*

Authentication: *Describe any authentication procedures for each seed stock used or novel genotype generated. Describe any experiments used to assess the effect of a mutation and, where applicable, how potential secondary effects (e.g. second site T-DNA insertions, mosiacism, off-target gene editing) were examined.*
